# Strain-Promoted Reaction of 1,2,4-Triazines with Bicyclononynes

**DOI:** 10.1002/chem.201502397

**Published:** 2015-08-13

**Authors:** Katherine A Horner, Nathalie M Valette, Michael E Webb

**Affiliations:** School of Chemistry and Astbury Centre for Structural Molecular Biology, University of Leeds Woodhouse Lane, LS2 9JT Leeds (UK) E-mail: m.e.webb@leeds.ac.uk

**Keywords:** bioorthogonal chemistry, chemical biology, strain-promoted reactions, synthetic methods, triazines

## Abstract

Strain-promoted inverse electron-demand Diels–Alder cycloaddition (SPIEDAC) reactions between 1,2,4,5-tetrazines and strained dienophiles, such as bicyclononynes, are among the fastest bioorthogonal reactions. However, the synthesis of 1,2,4,5-tetrazines is complex and can involve volatile reagents. 1,2,4-Triazines also undergo cycloaddition reactions with acyclic and unstrained dienophiles at elevated temperatures, but their reaction with strained alkynes has not been described. We postulated that 1,2,4-triazines would react with strained alkynes at low temperatures and therefore provide an alternative to the tetrazine cycloaddition reaction for use in in vitro or in vivo labelling experiments. We describe the synthesis of a 1,2,4-triazin-3-ylalanine derivative fully compatible with the fluorenylmethyloxycarbonyl (Fmoc) strategy for peptide synthesis and demonstrate its reaction with strained bicyclononynes at 37 °C with rates comparable to the reaction of azides with the same substrates. The synthetic route to triazinylalanine is readily adaptable to late-stage functionalization of other probe molecules, and the 1,2,4-triazine-SPIEDAC therefore has potential as an alternative to tetrazine cycloaddition for applications in cellular and biochemical studies.

## Introduction

**Scheme 1:**
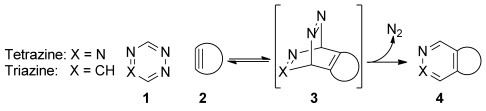
SPIEDAC between a tetrazine (1, X=N) or a triazine (1, X=CH) and a strained cycloalkyne 2. Rate-determining [4+2] cycloaddition between 1 and 2 led to the highly strained bicyclic adduct 3, which undergoes a cycloreversion yielding pyridazine (4, X=N) or pyridine-(4, X=CH) products and releasing dinitrogen.

Strain-promoted inverse electron-demand Diels–Alder cycloaddition (SPIEDAC) between 1,2,4,5-tetrazines and strained alkenes and alkynes are the fastest known bioorthogonal conjugation reaction. For example, tetrazines (Scheme 1, X=N) react with strained cycloalkynes to form pyridazine products via a sequential inverse electron-demand hetero-Diels–Alder (ihDA) retro-Diels–Alder (rDA) cascade.[1] Because this class of reactions proceeds with rate constants ranging from 1–10^5^ m^−1^ s^−1[2, 3]^ and gives no toxic by-products, they have become the reaction of choice for in vivo studies.[4–8] This class of cycloaddition has now been applied in multiple applications including intracellular imaging,[4] in vivo imaging,[6, 8] live labelling of cell-surface antigens,[7] as well as the modification of cells with nanomaterials for clinical diagnostics.[5]

Although tetrazine SPIEDAC reactions are rapid and efficient, production of functionalised tetrazine scaffolds remains synthetically challenging in comparison to triazine alternatives. Unsymmetrical aromatic and aliphatic tetrazines can either be accessed inefficiently through the Pinner synthesis,[2, 9] or via S_N_Ar reaction of a preformed symmetrical tetrazine, and a corresponding multistep synthesis.[10–14] The more efficient optimised routes to substituted tetrazines involve volatile precursors and intermediates; for example, the use of anhydrous hydrazine in the metal-mediated synthesis of aliphatic tetrazines described by Yang et al.,[15] or the “highly energetic” 3,6-dihydrazino-1,2,4,5-tetrazine intermediate in the synthesis of 3,6-dichloro-1,2,4,5-tetrazine.[14] In some cases, the tetrazine derivatives required for late-stage functionalisation are readily decomposed, for example, 3,6-dimethyldicarboxylate-1,2,4,5-tetrazine is prone to acid-promoted rearrangement and slowly decomposes upon warming[16] and 3,6-dimethylthio- and 3,6-dichlorotetrazines are incompatible with organometallic species.[17] Furthermore, some tetrazines are prone to either hydrolysis[9] or decomposition into the corresponding pyrazoles or thiazoles when exposed to endogenous cellular nucleophiles.[18]

Cycloaddition reactions have also been reported to occur between 1,2,4-triazines (Scheme 1, X=CH) and dienophiles to form dihydropyridine and pyridine derivatives.[19, 20] The earliest examples of 1,2,4-triazine cycloaddition reactions involved their conjugation to simple nitriles.[21] More recently, attention has been focused on exploiting the reaction to access a range of polycyclic and fused heterocycles through tethered triazine alkyne/alkene scaffolds.[20, 22] Although this cycloaddition is now commonly used in elegant synthetic routes to provide complex pyridyl-containing structures, the need for elevated temperatures (mostly exceeding 100 °C) and extended reaction times means that it has never been considered for cellular applications. 1,2,4-Triazine and 1,2,4,5-tetrazine conjugations are controlled by the HOMO_dienophile_–LUMO_diene_ gap; dienophiles with a high degree of ring strain reduce the activation energy of the [4+2]cycloaddition RDS by raising the HOMO_dienophile_ and decreasing the distortion energy needed to reach the cycloaddition transition state.[23, 24] To date, nearly all examples of 1,2,4-triazine cycloaddition reactions involve open chain and unstrained cyclic dienophiles. Until recently,[25] there were no reports of a 1,2,4-triazine SPIEDAC reaction with strained dienophiles other than norbornadiene.[26, 27]

**Scheme 2:**
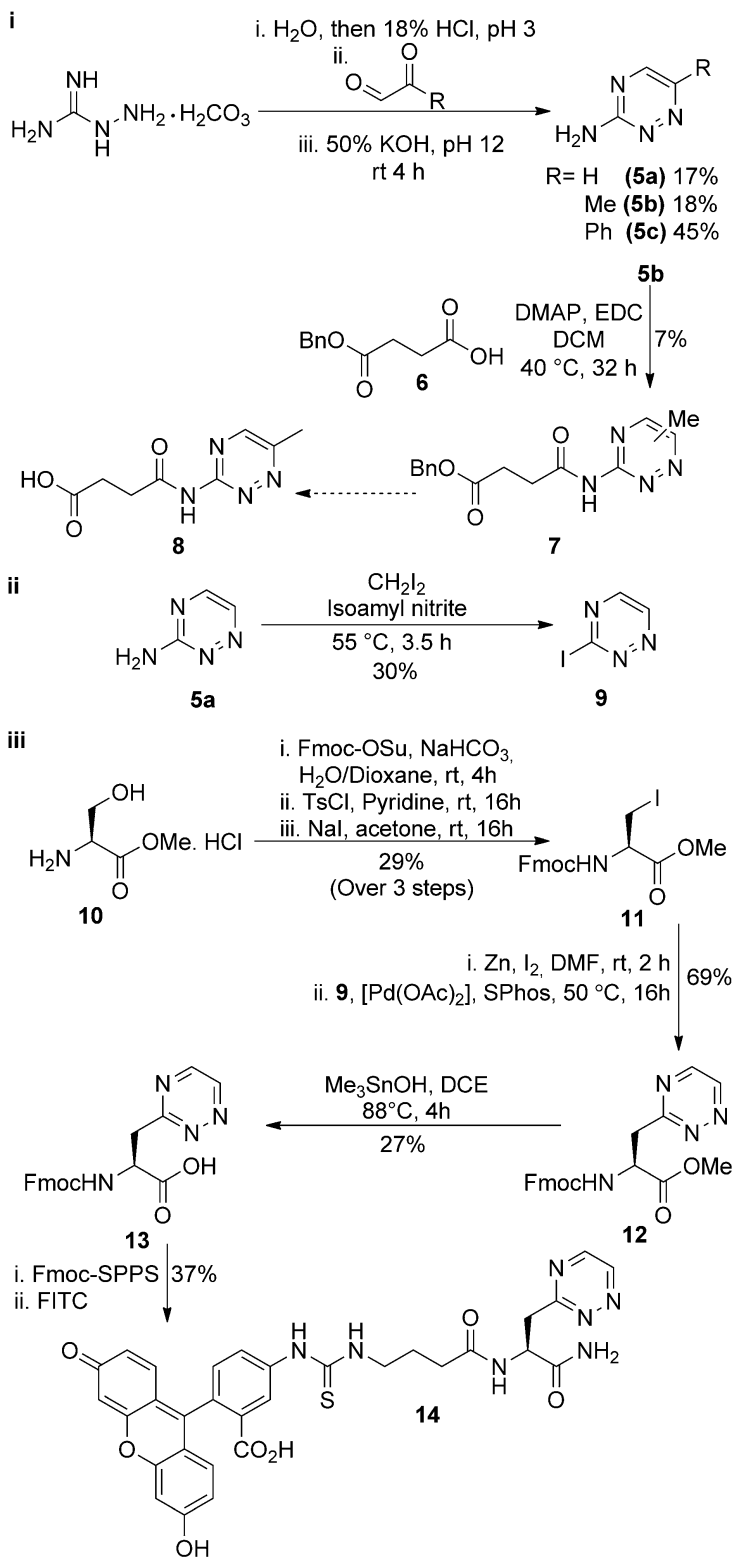
i) Condensation of aminoguanidine and a variety of substituted glyoxal derivatives to make substituted 3-amino-1,2,4-triazines 5 a–c and subsequent conversion in low yield to amide linked scaffolds. ii) Conversion of commercial 3-amino-1,2,4-triazine 5 a to 3-iodo-1,2,4-triazine 9 by direct iodination. iii) Synthetic route from serine methyl ester 10 to Fmoc-compatible building block Fmoc-TrzAla-OH 13 and subsequent incorporation into a peptide.

Based on the understanding that strained dienophiles increase the reaction rate for cycloaddition with 1,2,4,5-tetrazines, we proposed that the conjugation of a strained cycloalkane/alkyne with a 1,2,4-triazine derivative would also proceed without the need for elevated temperatures, and could thus offer an alternative to 1,2,4,5-tetrazine SPIEDAC for use in a range of in vitro and in vivo applications. We envisaged that this alternative, although slower, would obviate the need for toxic and volatile precursors and increase the range of probe molecules that could be generated. Herein, we report the synthesis of a new 1,2,4-triazinylalanine (TrzAla) derivative **13** (Scheme 2 iii), which is compatible with the fluorenylmethyloxycarbonyl (Fmoc) solid-phase peptide synthesis (SPPS) strategy, demonstrate its incorporation into a model probe peptide **14** and determine the rate of reaction of these compounds to a representative dienophile bicyclononyne **19**. This reaction has the potential for direct application for conjugation to genetically incorporated bicyclononyne-containing amino acids in future applications.

## Results and Discussion

Our initial aim was to synthesise a triazine with a functional group handle suitable for rapid derivatisation of target molecules. We initially synthesised 6-substituted 3-amino-1,2,4-triazines **5 a**–**c** (Scheme 2 i) with the aim of generating molecules of the form **8** via amide bond coupling. However, the low nucleophilicity of the exocyclic amine meant that we were able to generate the protected succinylamidotriazine **7** in only 1 % yield over two steps. In general, the de novo synthesis of triazines by controlled condensation between aminoguanidine and glyoxal derivatives (Scheme 2 i) is low yielding and gives mixtures of isomers; however, the aminotriazine derivative **5 a** is now commercially available in large quantities, and we therefore used this as the basis for subsequent reactions (Scheme 2 ii).

Jackson and co-workers have reported the synthesis of enantiomerically pure pyridylalanine amino acids through palladium-catalysed cross-coupling of serine-derived organozinc reagents with halopyridyl derivatives.[28, 29] We postulated that Negishi-type cross-coupling could be extended to include other N-heterocycles, and therefore devised a synthetic strategy (Scheme 2 iii) that utilised iodotriazine **9** as the electrophilic coupling partner. Iodotriazine **9** was formed through diazotisation of aminotriazine with isopentyl nitrite in diiodomethane (Scheme 2ii).[30] Fmoc-iodoalanine methyl ester **11** was synthesised in three steps from serine methyl ester **10**.[28]

For Negishi cross-coupling, commercial zinc dust was activated with iodine in anhydrous DMF. The use of a dipolar aprotic solvent, such as DMF prevents coordination of the carbamate carbonyl group to zinc, and therefore promotes β-elimination (by coordination to the carbonyl methyl ester) to form the organozinc reagent.[31] Zinc insertion of iodoalanine methyl ester **11** was complete after two hours at room temperature (determined by loss of the iodoalanine by TLC), and the subsequent cross-coupling with iodotriazine **9** was performed using catalytic amounts of palladium(II) acetate and 2-dicyclohexylphosphino-2′,6′-dimethoxybiphenyl (SPhos). This gave the desired triazinylalanine methyl ester **12** in a yield of 69 %. Limited attempts to optimise this coupling by increased catalyst loading and the alternative palladium catalyst (tris(dibenzylideneacetone)dipalladium(0)) did not lead to significant increases in yield. Demethylation of the methyl ester **12** to give the free acid **13** was carried out using trimethyltin hydroxide[32] in a yield of 27 %. In this case, the overall isolated yield is limited by observable degradation of the triazines **12** and **13** at the elevated temperatures required for deprotection. (Note that base-catalysed deprotection with LiOH leads to concomitant Fmoc-group removal.) To demonstrate the compatibility of the protected triazinylalanine with the conditions for the Fmoc strategy for solid-phase peptide synthesis, we generated a simple fluorescein isothiocyanate (FITC)-labelled peptide **14** using Rink amide resin and on-resin fluorescence labelling in 37 % yield following HPLC purification.

**Scheme 3:**
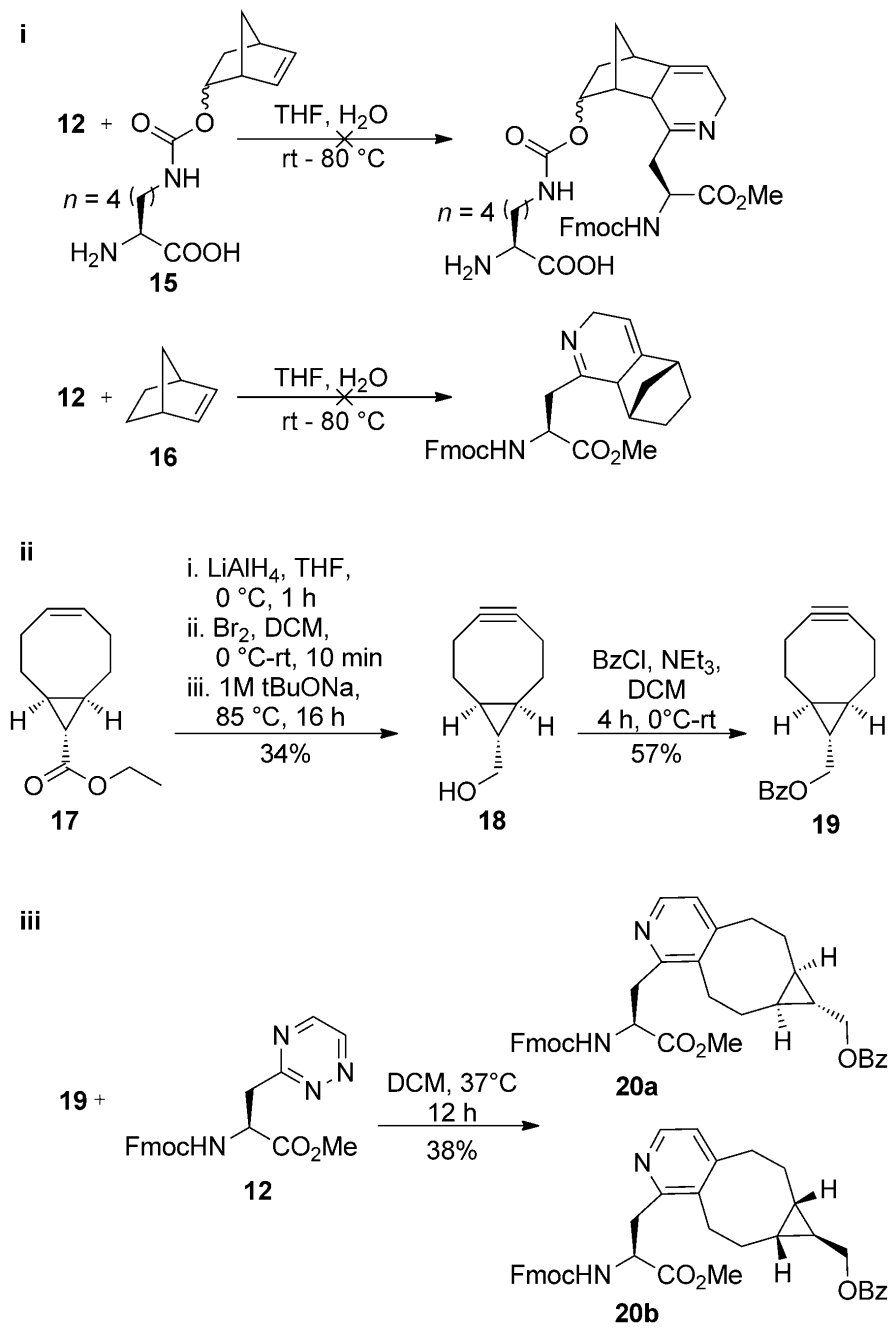
i) Attempted reaction of triazinylalanine methyl ester 12 with norbornene derivatives 15 and 16 led to no observable product formation. ii) Synthesis of benzoylated bicyclononyne 19 from single diastereoisomer of bicyclononene 17. iii) Reaction of protected Fmoc-TrzAla-Ome 12 with protected bicyclononylyl benzoate 19 gave a mixture of diastereoisomers 20 a and b at 37 °C.

We next evaluated suitable cycloaddition partners to couple to our 1,2,4-triazine derivatives **12** and **13**. There are a number of strained cyclic dienophiles reported for tetrazine SPIEDAC conjugations (with rate constants spanning five orders of magnitude).[2, 3] We initially investigated the cycloaddition reaction with norbornene, which reacts with tetrazine with a relatively slow rate constant (1–10 m^−1^ s^−1^).[2] We used the methyl ester **12** for rate determination to avoid potential interference from the free acid in **13**. We first synthesised norbornenyl-lysine **15** according to the procedure of Lang et al.[2], but could not detect formation of reaction product between **12** and **15** following prolonged incubation up to 80 °C (below the temperature at which we had observed thermal degradation of the triazines; Scheme 3 i). To ensure that the unprotected norbornenyl-lysine **15** was not interfering with our analysis, we confirmed that unfunctionalised norbornene **16** also did not react under these conditions.

The unhindered, strained bicyclo[6.1.0]nonynes react more rapidly with tetrazines with approximate rate constants of 10^2.5^–10^3.5^ m^−1^ s^−1^;[3] only exceeded by the rates of reaction to *trans*-cyclooctenes. We generated benzoyl-protected bicyclononyne **19** (Scheme 3 ii) by adaptation of the synthetic route of Dommerholt et al.;[33] following rhodium-catalysed cyclopropanation of 1,5-cyclooctadiene to yield a mixture of the *anti-* and *syn-*bicyclononene ethyl esters **17** and **17 b**, *anti-*bicyclononene ethyl ester **17** was converted to bicyclononylol **18** (Scheme 3 ii) by sequential reduction, dibromination and double elimination, followed by protection using benzoyl chloride to give alkyne **19**.[34]

**Figure 1:**
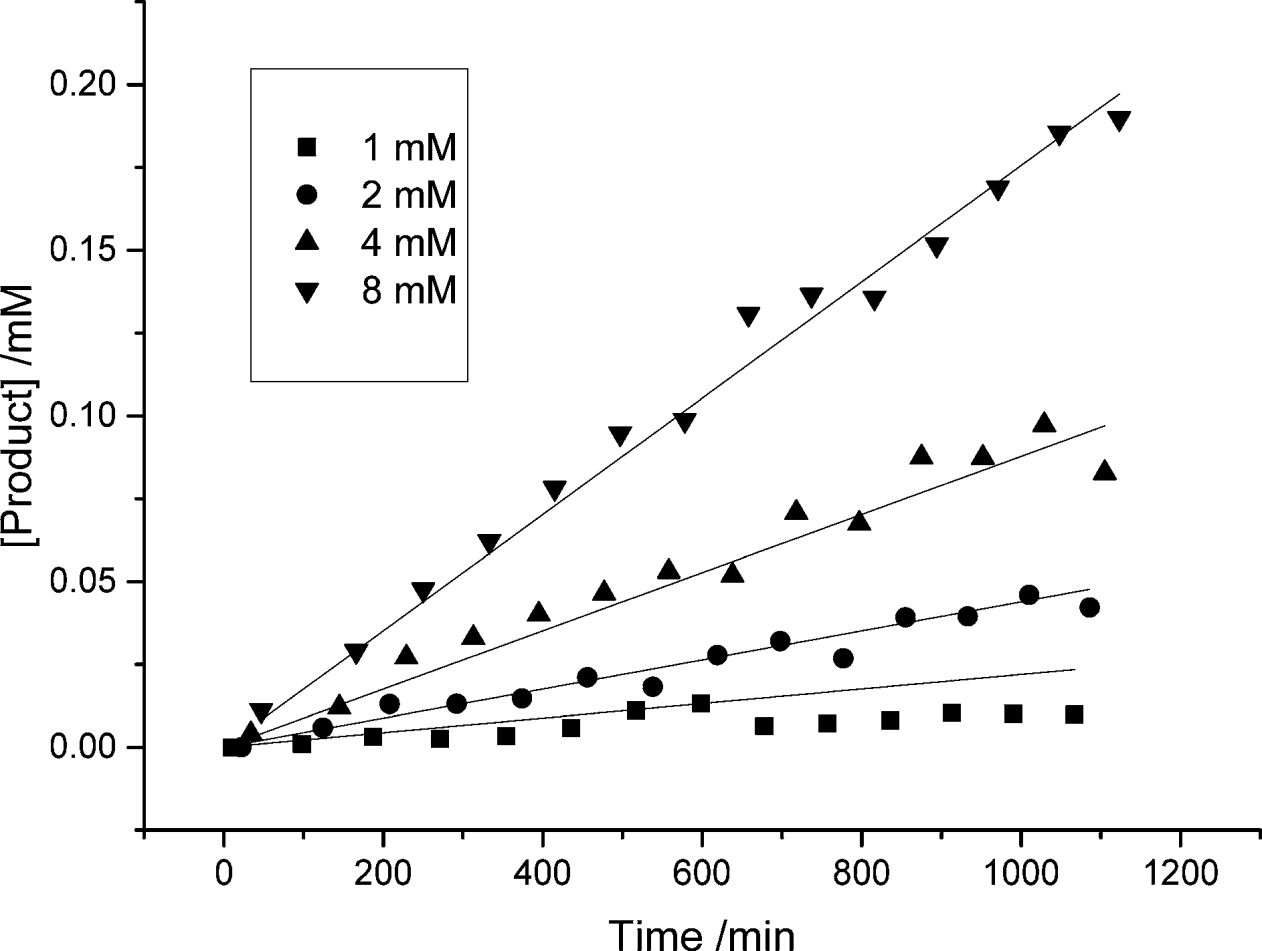
Rate of reaction of triazinylalanine methyl ester (TrzAla) 12 (1 mm) with bicyclononyne (BCN) 19 determined by HPLC measurement of formation of products 20 a and b. Rate data were globally fitted to second-order kinetics (rate=*k*_2_[BCN][TrzAla]) under the assumption of low substrate consumption during the measured time course.

We initially assessed the reaction of the protected bicyclononyne **19** with **12** at high concentration (65 mm in dichloromethane). Incubation of an equimolar mixture of the two reaction components yielded a 1:1 mixture of the bicyclononapyridyl derivatives **20 a** and **b** in 38 % yield after 12 h at 37 °C together with unreacted triazine **12** (Scheme 3 iii). In this case, the reaction was limited by apparent degradation of the bicyclononyne coupling partner (as was determined by NMR). Next, we assessed the reaction rate at lower concentrations by determining the rate of product formation by HPLC, using the purified mixture of authentic **20 a** and **b** as a concentration standard (Figure 1). Product formation in MeCN was measured over approximately 18 h using 1 mm **12** and increasing concentrations of the bicyclononyne **19**. Global fitting of the data gave an estimate for the second-order rate constant *k*_2_ of 2.30±0.03 ×10^−2^ m^−1^ min^−1^. Compounds **12** and **19** are poorly water soluble, preventing us from carrying out the analogous experiment in water; however, the reaction rate in 10 % H_2_O/MeCN (see the Supporting Information) increased slightly to 3.0±0.03 ×10^−2^ m^−1^ min^−1^ suggesting that the rate in biological media will be slightly higher, consistent with other examples of this class of reaction.[35, 36]

Comparison of this rate of reaction (0.3–0.5×10^−3^ m^−1^ s^−1^) to other reactions in this class[37] suggests that although the cycloaddition of triazines with bicyclononynes is much slower than the corresponding reaction of tetrazines, it is comparable with other known bioorthogonal reactions, such as the Staudinger ligation. With suitable reaction partners, it has the potential to have comparable rates to the strain-promoted addition of azides to alkynes. Very recently, Kamber et al. have reported a complementary study of the reaction between 1,2,4-triazin-6-yl derivatives and strained *trans*-cyclooctenes.[25] Over a range of triazine substrates, they observe reaction rates of between 1 and 7×10^−2^ m^−1^ s^−1^—approximately 30-fold higher than those we have determined. This ratio is similar to the approximately 15-fold difference in rate observed for the reaction of tetrazines with bicyclononyne and *trans*-cyclooctene substrates by Lang et al.[3] and Kamber et al. observations are therefore fully consistent with our observed reaction rates.

## Conclusion

We have defined a route to 1,2,4-triazin-3-yl-linked amino acids compatible with conventional peptide-synthesis strategies using readily available and inexpensive starting materials as precursors. The alkyl triazine reacts readily with the strained bicyclononyne dienophile at 37 °C indicating that it is suitable for protein-labelling applications. The synthetic strategy adopted can be readily adapted to generate triazine-linked scaffolds at a late stage. The amino acid is similar in structure to a range of tyrosine-based scaffolds that have been genetically incorporated into proteins in response to an amber codon using evolved tyrosyl-tRNA synthetases.[38, 39] We hypothesise that that it will be possible to identify such systems as has been recently demonstrated for triazinylphenylalanine by Kamber et al.[25] But because strategies to incorporate bicyclononyne-containing amino acids into proteins are already established,[40] this is not a limiting factor for application to site-specific labelling in an in vitro or in vivo context.

## Experimental Section

General chemical experimental details procedures for synthesis of compounds **5 a**, **b**, **c**, **6**, **7**, **10**, **11**, **14**, **17 a**, **18** and **19**, and protocols for rate determination can be found in the Supporting Information.

### Synthesis of 3-iodo-1,2,4-triazine (9)

Isoamyl nitrite (42 mL, 300 mmol, 14 equiv) was added to a stirred solution of 3-amino-1,2,4-triazine **5 a** (2 g, 20 mmol, 1 equiv) in diiodomethane (ca. 40 mL).[30] The turbid orange mixture was stirred at 55 °C for 4 h, allowed to cool, filtered at the pump and concentrated as far as possible in vacuo to leave the product and residual unreacted diiodomethane (ca. 30 mL). The remaining filtrate was applied to a silica column and purified by column chromatography eluting with 3:1 hexanes/EtOAc. The resultant orange solid was dissolved in 1,4-dioxane and lyophilised to give the product (1.25 g, 6.04 mmol, 30 %) as a flocculent orange solid. *R*_F_ (3:1 hexanes/EtOAc) 0.29; ^1^H NMR (500 MHz, CDCl_3_): *δ*=9.26 (1 H, d, ^3^*J*_H−H_ 2.1 Hz, H_6_), 8.38 ppm (1 H, d, ^3^*J*_H−H_ 2.2 Hz, H_5_); ^13^C NMR (125 MHz, CDCl_3_): *δ*=149.20 (C_5_), 148.37 ppm (C_6_); 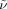
_max_ (solid): 3450, 3417 cm^−1^ (NH_2_ stretch); MS (ES): *m/z* calcd for C_3_H_3_IN_3_: 207.9293 [*M*+H]; found: 207.9366; HPLC (5–95 % A): retention time 1.41 min, 100 %.

### Synthesis of Fmoc-TrzAla-OMe (9*H*-fluoren-9-yl)methyl (*S*)-1-(methoxycarbonyl)-2-(1,2,4-triazin-3-yl)ethylcarbamate (12)

To an oven-dried two-neck flask, zinc dust (1.16 g, 18 mmol, 3 equiv) was added; the flask was evacuated, dried with a flame and purged with nitrogen three times.[28] The flask was allowed to cool to room temperature, dry DMF (18 mL) and iodine (225 mg, 0.89 mmol, 0.15 equiv) were added in quick succession. The solution became orange, and after two minutes returned to grey. After 15 min, iodoalanine **11** (2.67 g, 5.9 mmol, 1 equiv) was added, followed immediately by iodine addition (225 mg, 0.89 mmol, 0.15 equiv), and the mixture was stirred at room temperature. After two hours, zinc activation was shown to be complete by TLC (2:1 hexanes/EtOAc) and 3-iodo-1,2,4-triazine (**9**; 1.59 g, 7.68 mmol, 1.3 equiv), palladium(II) acetate (33 mg, 0.15 mmol, 0.025 equiv) and 2-dicyclohexylphosphino-2′,6′-dimethoxybiphenyl (SPhos) (121 mg, 0.30 mmol, 0.05 equiv) were added to the flask in quick succession. The flask was heated to 50 °C and stirred for five hours, the reaction was allowed to cool and filtered through a Celite pad, which was washed several times with CH_2_Cl_2_. The resultant solution was concentrated in vacuo, and the pale orange solid was purified by column chromatography on silica gel, eluting initially with 4:1 hexanes/EtOAc and then EtOAc. The combined fractions were concentrated in vacuo and lyophilised to give Fmoc-TrzAla-OMe (1.66 g, 4.12 mmol, 69 %) as a flocculent, pale orange solid. [*α*]
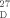
=+4.12 (*c=*0.19, CH_2_Cl_2_); *R*_F_ (EtOAc) 0.72; ^1^H NMR (500 MHz, CDCl_3_): *δ*=9.36 (1 H, s, Tz-*H*_6_), 8.81 (1 H, d, ^3^*J*_H−H_ 2.4, Tz-*H*_5_), 7.79–7.73 (2 H, m, Fmoc-*H*_4_), 7.58 (2 H, t, ^3^*J*_H−H_ 7.4, Fmoc-*H*_1_), 7.43–7.37 (2 H, m, Fmoc-*H*_3_), 7.31 (2 H, t, ^3^*J*_H−H_ 7.3, Fmoc-*H*_2_), 5.98 (1 H, d, ^3^*J*_H−H_ 8.5, N*H*), 5.07–4.96 (1 H, m, *H*_α_), 4.45–4.35 (2 H, m, CHC*H_2_*), 4.21 (1 H, t, ^3^*J*_H−H_ 6.97, C*H*CH_2_), 3.79–3.72 ppm (5 H, m, *H*_β_ and OC*H_3_*); ^13^C NMR (125 MHz, CDCl_3_): *δ*=171.7 (*C*O_2_Me) 166.4 (Tz-C_3_), 155.8 (OCO.NH), 151.9 (Tz-*C*_*5/6*_), 147.4 (Tz-*C*_*5/6*_), 143.7 (Fmoc-*C*_5/6_), 140.7 (Fmoc-*C*_5/6_) 127.8 (Fmoc-*C*_3_), 127.1 (Fmoc-*C*_2_), 125.2 (Fmoc-*C*_1_), 120.0 (Fmoc-*C*_4_), 67.4 (*C*_β_), 67.1 (CH*C*H_2_), 53.1 (*C*_α_), 51.9 (O*C*H_3_), 47.0 ppm (*C*HCH_3_); 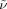
_max_ (solid): 3049 and 2950 cm^−1^ (NH stretch), 1715 (CO); MS (ES): *m/z* calcd for C_22_H_21_N_4_O_4_: 405.1557 [*M*+H]; found: 405.1560; HPLC (5–95 % B): retention time 2.93 min, 100 %.

### Synthesis of Fmoc-TrzAla-OH (9*H*-fluoren-9-yl)methyl (*S*)-1-(carboxy)-2-(1,2,4-triazin-3-yl)ethylcarbamate (13)

Fmoc-TrzAla-OMe (**12**; 300 mg, 0.74 mmol, 1 equiv) and trimethyltin hydroxide (402 mg, 2.2 mmol, 3 equiv) were dissolved in anhydrous C_2_H_4_Cl_2_ (9 mL) and heated at reflux for 2.5 h, until the deprotection was shown to be complete by TLC (EtOAc).[32] The reaction was allowed to cool to room temperature and quenched with H_2_O (15 mL). The organic layer was extracted with CH_2_Cl_2_ (3×20 mL), and the combined organic layers were washed with H_2_O (1×20 mL), brine (2×20 mL), dried (MgSO_4_) and concentrated in vacuo. The orange oil was purified by column chromatography on silica gel (95:4:1 CH_2_Cl_2_/MeOH/AcOH) and lyophilised to give Fmoc-trzAla-OH (78 mg, 0.19 mmol, 27 %) as a pale yellow, flocculent solid. [*α*]
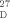
=+4.90 (*c=*0.10, CH_2_Cl_2_); *R*_F_ (CH_2_Cl_2_/MeOH/AcOH) 0.26; ^1^H NMR (500 MHz, CDCl_3_): *δ*=9.18 (1 H, s, Tz-*H*_6_), 8.63 (1 H, s, Tz-*H*_5_), 7.75 (2 H, d, ^3^*J*_H−H_ 7.5, Fmoc-*H*_4_), 7.57 (2 H, dd, ^3^*J*_H−H_ 7.4, ^4^*J*_H−H_ 3.1, Fmoc-*H*_1_), 7.39 (2 H, t, ^3^*J*_H−H_ 7.3, Fmoc-*H*_3_), 7.30 (2 H, t, ^3^*J*_H−H_ 7.4, Fmoc-*H*_2_), 6.05 (1 H, d, ^3^*J*_H−H_ 8.3. N*H*), 5.04–4.97 (1 H, m, *H*_α_), 4.39 (2 H, dd, ^2^*J*_H−H_ 17.1, ^3^*J*_H−H_ 9.1, CHC*H_2_*), 4.22 (1 H, t, ^3^*J*_H−H_ 7.1, C*H*CH_2_), 3.79 (1 H, d, ^3^*J*_H−H_ 5.5, *H*_*β*_), 3.76 ppm (1 H, d, ^3^*J*_H−H_ 5.0, *H*_*β*_); ^13^C NMR (125 MHz, CDCl_3_): *δ*=156.1 (O*C*ONH), 149.1 (Tz-*C*_5/6_), 148.0 (Tz-*C*_5/6_), 143.8 (Tz-*C*_3_), 143.7 (Fmoc-*C*_5/6_), 141.3 (Fmoc-*C*_5/6_) 127.7 (Fmoc-*C*_3_), 127.1 (Fmoc-*C*_2_), 125.1 (Fmoc-*C*_1_), 120.0 (Fmoc-*C*_4_), 67.3 (*C*_β_), 67.1 (CH*C*H_2_), 52.2 (*C*_α_), 39.0 ppm (*C*HCH_3_); 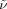
_max_ (solid): 3379 (OH stretch), 1714 cm^−1^ (CO); MS (ES): *m/z* calcd for C_21_H_19_N_4_O_4_: 391.1401 [*M*+H]; found: 391.1401; HPLC (5–95 % B): retention time 2.87 min, 100 %.

### Synthesis of methyl (2*S*)-3-[2-((1S*,8R*,9R*)-9-benzoyloxymethylbicyclo[6.1.0]nona [4,5-c]pyridyl)-2-((1*S*)*N*-(9-fluorenylmethoxycarbonyl)amino)propionate (20 a and b)

To a solution of (*Z*,1*S*,8*R*,9*r*)-bicyclo[6.1.0]non-4-ene-9-ylmethanol (**19**; 66 mg, 0.26 mmol, 1 equiv) in CH_2_Cl_2_ (2 mL), Fmoc-TrzAla-OMe (**12**; 105 mg, 0.26 mmol, 1 equiv) in CH_2_Cl_2_ (2 mL) was added, and the reaction was stirred at 37 °C for 16 h, after which time, complete consumption of (*Z*,1*S*,8*R*,9*r*)-bicyclo[6.1.0]non-4-ene-9-ylmethanol was observed (4:1 hexanes/EtOAc). The orange solution was concentrated in vacuo and purified by column chromatography on silica gel (5 % MeOH in CH_2_Cl_2_), the resultant pale yellow solid was dissolved in dioxane and lyophilised to give the product (62 mg, 0.09 mmol, 38 %) as a pale yellow flocculent solid. [*α*]
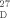
=+4.7 (*c=*0.11, CH_2_Cl_2_); *R*_F_ (25:1 hexanes/EtOAc) 0.09; ^1^H NMR ([D_2_]CH_2_Cl_2_, 500 MHz); *δ*=8.26–8.18 (1 H, m, *H*_9_), 8.08–7.96 (2 H, m, Bz-*H*_2_), 7.79 (2 H, dd, ^3^*J*_H−H_ 7.6, ^4^*J*_H−H_ 4.0 Hz, Fmoc-*H*_4_), 7.67–7.55 (3 H, m, Bz-*H_4_* and Fmoc-*H_1_*), 7.53–7.43 (2 H, m, Bz-*H*_3_), 7.43–7.38 (2 H, m, Fmoc-*H*_3_), 7.37–7.28 (2 H, m, Fmoc-*H*_2_), 7.03–6.93 (1 H, m, *H*_8_), 6.64–6.55 (1 H, m, N*H*), 4.83–4.73 (1 H, m, *H*_α_), 4.43–4.29 (2 H, m, Fmoc-CHC*H_2_*), 4.25 (1 H, t, *J*=7.1 Hz, C*H*CH_2_), 4.12–3.98 (2 H, m, Bz-COOC*H*_2_), 3.54 (1 H, ddd, ^2^*J*_H−H_ 17.0, ^4^*J*_H−H_ 12.1, ^3^*J*_H−H_ 5.6 Hz, *H*_β_), 3.31 (1 H, ddd, ^2^*J*_H−H_ 16.0, ^4^*J*_H−H_ 11.4, ^3^*J*_H−H_ 4.3 Hz, *H*_β_), 3.05–2.92 (2 H, m, *H*_3_), 2.87–2.72 (2 H, m, *H*_3’_), 2.64–2.44 (2 H, m, *H*_4_), 1.48–1.33 (2 H, m, *H*_4’_), 0.95–0.88 (1 H, m, *H*_5_), 0.85–0.70 ppm (2 H, m, *H*_4a_); ^13^C NMR (125 MHz, CDCl_3_): *δ*=172.8 (*C*OOCH_3_), 166.8 (Bz-*C*OCH_2_), 156.5 (Fmoc-*C*OONH), 155.0 (*C*_2_), 152.3 (*C*_2a_), 146.1 (*C*_9_), 144.4 (Fmoc-*C*_4a_), 141.6 (Fmoc-*C*_1a_), 136.6 (*C*_7a_), 133.1 (Fmoc-*C*_1_), 131.0 (Bz-*C*_1_), 129.8 (Bz-*C*_2_), 128.7 (Bz-*C*_3_), 128.0 (Fmoc-*C*_3_), 127.4 (Fmoc-*C*_2_), 125.5 (Bz-*C*_4_), 124.5 (*C*_8_), 120.3 (Fmoc-*C*_4_), 68.8 (*C*_5’_), 67.2 (Fmoc-CH*C*H_2_), 53.1 (COO*C*H_3_), 52.5 (*C*_α_), 47.6 (Fmoc-*C*HCH_2_), 35.9 (*C*_β_), 33.9 (*C*_3_), 29.0 (*C*_4_), 26.7 (*C*_5_), 22.4 (*C*_4a_); 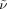
_max_ (solid): 3335 (NH stretch), 1714 cm^−1^ (CO); MS (ES): *m*/*z* calcd for C_39_H_39_N_2_O_6_: 631.2803 [*M*+H]; found 631.2814; HPLC (5–95 % B): 4.75 min, 100 %.

## Supporting Information

As a service to our authors and readers, this journal provides supporting information supplied by the authors. Such materials are peer reviewed and may be re-organized for online delivery, but are not copy-edited or typeset. Technical support issues arising from supporting information (other than missing files) should be addressed to the authors.

miscellaneous_information

## References

[1] Knall A-C, Slugovc C (2013). Chem. Soc. Rev.

[2] Lang K, Davis L, Torres-Kolbus J, Chou C, Deiters A, Chin JW (2012). Nat. Chem.

[3] Lang K, Davis L, Wallace S, Mahesh M, Cox DJ, Blackman ML, Fox JM, Chin JW (2012). J. Am. Chem. Soc.

[4] Devaraj NK, Hilderbrand S, Upadhyay R, Mazitschek R, Weissleder R (2010). Angew. Chem. Int. Ed.

[b01] (2010). Angew. Chem.

[5] Haun JB, Devaraj NK, Hilderbrand SA, Lee H, Weissleder R (2010). Nat. Nanotechnol.

[6] Rossin R, Verkerk PR, van den Bosch SM, Vulders RCM, Verel I, Lub J, Robillard MS (2010). Angew. Chem. Int. Ed.

[b02] (2010). Angew. Chem.

[7] Devaraj NK, Upadhyay R, Hatin JB, Hilderbrand SA, Weissleder R (2009). Angew. Chem. Int. Ed.

[8] Laughlin ST, Baskin JM, Amacher SL, Bertozzi CR (2008). Science.

[9] Karver MR, Weissleder R, Hilderbrand SA (2011). Bioconjugate Chem.

[10] Boger DL, Sakya SM (1988). J. Org. Chem.

[11] Boger DL, Schaum RP, Garbaccio RM (1998). J. Org. Chem.

[12] Gong Y-H, Miomandre F, Méallet-Renault R, Badré S, Galmiche L, Tang J, Audebert P, Clavier G (2009). Eur. J. Org. Chem.

[13] Coburn MD, Buntain GA, Harris BW, Hiskey MA, Lee K-Y, Ott DG (1991). J. Heterocycl. Chem.

[14] Chavez DE, Hiskey MA (1999). J. Energ. Mater.

[15] Yang J, Karver MR, Li W, Sahu S, Devaraj NK (2012). Angew. Chem. Int. Ed.

[b03] (2012). Angew. Chem.

[16] Boger DL, Coleman RS, Panek JS, Huber FX, Sauer J (1985). J. Org. Chem.

[17] Clavier G, Audebert P (2010). Chem. Rev.

[18] Liu DS, Tangpeerachaikul A, Selvaraj R, Taylor MT, Fox JM, Ting AY (2012). J. Am. Chem. Soc.

[19] Balcar J, Chrisam G, Huber FX, Sauer J (1983). Tetrahedron Lett.

[20] Lahue BR, Lo S-M, Wan Z-K, Woo GHC, Snyder JK (2004). J. Org. Chem.

[21] Taylor EC, French LG (1989). J. Org. Chem.

[22] Foster RAA, Willis MC (2013). Chem. Soc. Rev.

[23] Thalhammer F, Wallfahrer U, Sauer J (1990). Tetrahedron Lett.

[24] Sauer J, Heldmann DK, Hetzenegger J, Krauthan J, Sichert H, Schuster J (1998). Eur. J. Org. Chem.

[26] Kozhevnikov VN, Kozhevnikov DN, Shabunina OV, Rusinov VL, Chupakhin ON (2005). Tetrahedron Lett.

[27] Shi B, Lewis W, Campbell IB, Moody CJ (2009). Org. Lett.

[28] Tabanella S, Valancogne I, Jackson RFW (2003). Org. Biomol. Chem.

[29] Jackson RFW, Wishart N, Wood A, James K, Wythes MJ (1992). J. Org. Chem.

[30] Carroll FI, Kotturi SV, Navarro HA, Mascarella SW, Gilmour BP, Smith FL, Gabra BH, Dewey WL (2007). J. Med. Chem.

[31] Jackson RFW, Moore RJ, Dexter CS, Elliott J, Mowbray CE (1998). J. Org. Chem.

[32] Furlán RLE, Mata EG, Mascaretti OA (1998). J. Chem. Soc. Perkin Trans. 1.

[33] Dommerholt J, Schmidt S, Temming R, Hendriks LJA, Rutjes FPJT, van Hest JCM, Lefeber DJ, Friedl P, van Delft FL (2010). Angew. Chem. Int. Ed.

[b04] (2010). Angew. Chem.

[34] Cruchter T, Harms K, Meggers E (2013). Chemistry.

[35] Blackman ML, Royzen M, Fox JM (2008). J. Am. Chem. Soc.

[36] Wijnen JW, Zavarise S, Engberts JBFN, Charton M (1996). J. Org. Chem.

[37] Lang K, Chin JW (2014). Chem. Rev.

[38] Wang L, Zhang Z, Brock A, Schultz PG (2003). Proc. Natl. Acad. Sci. USA.

[39] Xie J, Schultz PG (2005). Methods.

[40] Borrmann A, Milles S, Plass T, Dommerholt J, Verkade JMM, Wiessler M, Schultz C, van Hest JCM, van Delft FL, Lemke EA (2012). Chembiochem.

