# Supplementary material for: Strain-Promoted Reaction of 1,2,4-Triazines with Bicyclononynes
Source: Chemistry. 2015 Aug 13;21(41):14376–81. doi: 10.1002/chem.201502397 (PMC4600244; doi:10.1002/chem.201502397)
Supplement: Supplementary file 1 — miscellaneous_information [file chem0021-14376-sd1.pdf]

CHEMISTRY
A European Journal

Supporting Information

Strain-Promoted Reaction of 1,2,4-Triazines with Bicyclononynes

Katherine A. Horner, Nathalie M. Valette, and Michael E. Webb*[a]

chem_201502397_sm_miscellaneous_information.pdf

Supporting information

Time course of reaction between 19 and 13 in 1:9 (v/v) H_20/MeCN 2

General experimental 3

Synthesis of 5a-c, 6,7,Fmoc-Ser-OMe, Fmoc-Ser(OTs)-OMe, 11, 14, 17a, 18 & 19 4

General procedure for HPLC assay.. 12

NMR spectra, High-resolution mass spectra and HPLC chromatograms 14

Time course of reaction between 19 and 13 in 1:9 (v/v) H_20/MeCN

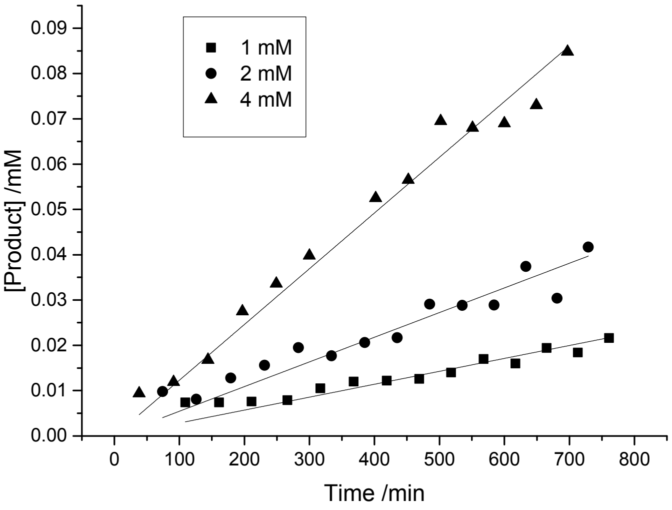

Figure S1 Rate of reaction of triazinylalanine (TrzAla) **13** (1 mM) with bicyclononyne (BCN) **19** in 10% H_20/MeCN determined by HPLC measurement of formation of products **20a** and **20b**. Rate data were globally fitted to 2nd order kinetics (Rate = k_2[BCN][TrzAla]) under the assumption of low substrate consumption during the measured time-course.

General experimental

All reagents were purchased from Sigma Aldrich, Alfa Aesar, Merck or Fisher Scientific and all were used without further purification. All solvents used were HPLC grade and mixtures are *v/v*. NMR data were collected using a Bruker DPX300 and analysed using MestReNova software. The following abbreviations are used to describe the multiplicity of signals: s = singlet, d = doublet, dd = doublet of doublets, qn = quintet, m = multiplet. J-values are given in Hz. IR spectra were recorded using a PerkinElmer spectrum one FTIR spectrometer, Vibrational frequencies are reported in wavenumbers (cm^{-1}). Optical rotations were measured using a Schmidt+Haensch polartronic H532, $[\alpha]_D$ values are given in 10^{-1} deg cm^2 g^{-1}. High resolution mass spectrometry (HRMS) was carried out on a Bruker Daltonics micrOTOF using electrospray ionisation or on a Micromass GCT Premier, using electron impact ionisation (EI). Column chromatography was carried out using silica gel and TLC was performed on silica gel 60-F_{254} (Merck) with detection by fluorescence upon irradiation with UV light or staining by immersion in a solution of potassium permanganate or ninhydrin and heating. HPLC analyses were carried out on a Dionex HPLC system using an Ace UltraCore 2.5 Super C18, (50 x 2.1mm) and diode array as a detector; either on gradient A: mobile phase (H_2O/MeCN, 0.1% TFA), gradient (5-95% MeCN) or B: mobile phase (H_2O/MeCN), gradient (5-95% MeCN), at a flow rate of 0.5 ml/min. Melting points were determined on a Stuart SMP3 melting point apparatus. Melting points obtained were uncorrected.

Synthesis of 5a-c, 6,7,Fmoc-Ser-OMe, Fmoc-Ser(OTs)-OMe, 11, 14, 17a, 18 & 19

3-Amino-1,2,4-triazine 5a

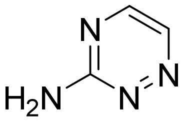

Aminoguanidine bicarbonate (3.00 g, 22 mmol, 1 eq) was stirred in H_2O (40 ml), the pH was dropped to 3-4 on addition of small portions of 18% HCl (~5 ml) until CO_2 gas evolution seized and the solution became clear.[43] Glyoxal (40% in H_2O) (2.5 ml, 22 mmol, 1 eq) was added and the pH increased to 12 with addition of 50% KOH (~5 ml). The mixture was stirred for 2 hours and lyophilised. The resultant brown powder was triturated in 1:3 hexanes/EtOAc and hot filtered. The solution was concentrated and the crude product was recrystallised from 1:3 hexanes/EtOAc to give the title compound (365 mg, 3.91 mmol, 17%) as pale orange needles. m.p. 179.2-179.9 ºC; R_F (5% MeOH in DCM) 0.17; δ_H (300 MHz, $CDCl_3$) 8.50 (1H, d, $^3J_{H-H}$ 2.4, H_6), 8.20 (1H, d, $^3J_{H-H}$ 2.4, H_5), 7.17 (2H, s, NH_2); δ_C (125 MHz, $CDCl_3$) 163.3 (C_3), 149.88 (C_5), 140.63 (C_6); ν_{max} (solid)/cm^{-1}: 3016 & 2970 (NH stretch); *m/z* (EI) Found: M(+H) 93.0, $C_3H_5N_4$ requires 93.0436); HPLC (5-95% A) retention time 0.43 min, 100%.

6-Methyl-3-amino-1,2,4-triazine and 5-methyl-3-amino-1,2,4-triazine 5b

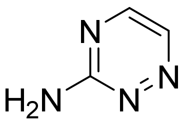

The reaction procedure was similar to that for **5a**. The Crude product was recrystallized twice from boiling isopropanol to give either a mixture of *6-Methyl-3-amino-1,2,4-triazine* and *5-methyl-3-amino-1,2,4-triazine* (2:1) (53.1 mg, 0.74 mmol, 18%) as a yellow solid or *6-Methyl-3-amino-1,2,4-triazine*: m.p. 122.0-125.6 ºC; R_F (5% MeOH in DCM) 0.17; δ_H (500 MHz,DMSO): 8.15 (1H, s, H_5), 6.90 (2H, s, NH_2), 2.38 (3H, s, CH_3), δ_C (125 MHz, $CDCl_3$): 162.0 (C_3), 150.2 (C_5), 147.8 (C_6), 17.9 (CH_3); ν_{max} (solid)/cm^{-1}: 3160 (NH_2); *m/z* (EI) Found: *M(+H)* 110.1 $C_4H_7N_4$ requires 110.0592. HPLC (5-95% A): retention time 1.11 min, 100%. (*6-methyl-3-amino-1,2,4-triazine* & *5-methyl-3-amino-1,2,4-triazine* (2:1): δ_H (500 MHz, MeOD): 8.46 (1H, s, 5-methyl-H_6), 8.23 (1H, s, 6-methyl-H_5), 2.48 (3H, s, 6-methyl-CH_3), (3H, s, 5-methyl-CH_3).

6-Phenyl-3-amino-1,2,4-triazine 5c

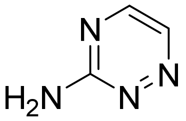

The reaction procedure was similar to that of **5a**. The crude product was recrystallized twice from boiling isoproponal to give *6-Phenyl-3-amino-1,2,4-triazine*

(100 mg, 0.58 mmol, 45%) as a flocculent orange solid; R_F (5% MeOH in DCM) 0.10;); δ_H (500 MHz, $CDCl_3$): 9.34 (1H, s, 5-H), 8.19 (2H, dd, $^3J_{H\text{-}H}$ 1.4, $^4J_{H\text{-}H}$ 8.2, benzyl-H_{2+5}), 7.63-7.56 (3H, m, benzyl-$H_{3\text{-}5}$), 7.25 (2H, s, NH_2); δ_C (125 MHz, $CDCl_3$): 163.8 (C_3), 150.4 (C_5), 135.7 (C_6), 130.3 (benzyl-C_1), 126.7 (benzyl-$C_{2\text{-}6}$); ν_{max} (solid)/cm^{-1}: 3301 (NH stretch); *m/z* (ES) Found: *M(+H)* 173.0822, $C_9H_9N_4$ requires 173.1866; HPLC: retention time 1.20 min, 90%.

3-((Benzyloxy)carbonyl)propanoic acid 6

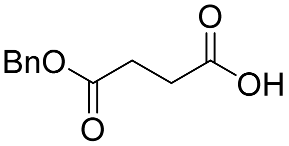

A mixture of succinic anhydride (2.00 g, 20 mmol, 1 eq), benzyl alcohol (2.1 ml, 20 mmol, 1 eq), DMAP (2.48 g, 20 mmol, 1 eq) and triethylamine (1 ml) in DCM (~30 ml) was stirred at room temperature for 16 hours.[44] The reaction was quenched with 5% Na_2CO_3 (~30 ml) and the layers were separated. The aqueous layer was acidified with 1M HCl (~pH 3-4), extracted with EtOAc (3 × 30 ml) and the combined organics washed with brine (2 × 20 ml), dried and evaporated to give the title compound *3-((Benzyloxy)carbonyl)propanoic acid* (2.52 g, 12.1 mmol, 61%) as a colourless amorphous solid; R_F (2:1 hexanes:EtOAc) 0.21; δ_H (500 MHz, $CDCl_3$): 7.39-7.26 (5H, m, Ar-*H*), 5.16 (2H, s, Ar-CH_2), 2.84-2.56 (4H, m, OCH_2CH_2O); δ_C (125 MHz, $CDCl_3$): 178.0 (*C*OOH), 172.0 (O*C*OCH$_2$), 135.7 (Ar-C_1), 128.6, 128.3 & 128.2 (Ar-$C_{2\text{-}6}$), 66.7 (Ar*C*H$_2$), 28.9 (O*C*H$_2$*C*H$_2$O); ν_{max} (solid)/cm^{-1}: 3181 (OH); 1736 (CO); *m/z* (ES): Found *M(+Na)* 231.0628. $C_{11}H_{12}O_4Na$ requires 231.0555); elemental analysis (Found: C, 62.5; H, 5.8; O, 30.7; $C_{11}H_{12}O_4$ requires C, 63.4; H, 5.8; O, 30.7); HPLC (5-95% A): retention time 2.14 min, 100%.

Benzyl 3-(6-methyl-1,2,4-triazin-3-ylcarbamoyl)propanoate and Benzyl 3-(5-methyl-1,2,4-triazin-3-ylcarbamoyl)propanoate 7

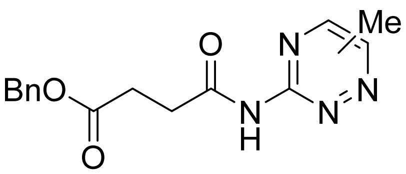

3-((benzyloxy)carbonyl)propanoic acid **6** (297 mg, 2.7 mmol, 1 eq), 5/6-methyl-1,2,4-triazin-3-amine **5b** (561 mg, 2.7 mmol, 1 eq), 4-dimethylaminopyridine (660 mg, 5.4 mmol, 2 eq) and 1-Ethyl-3-(3-dimethylaminopropyl)carbodiimide (1.03 g, 5.4 mmol, 2 eq) were stirred at 40°C under a nitrogen atmosphere in DMF (10 ml) for 32 hours. The reaction was quenched with H_2O (20 ml) and the aqueous layer extracted with EtOAc (4 × 30 ml). The combined organics were washed with H_2O (1 × 30 ml) and brine (2 × 40 ml), dried ($MgSO_4$) and concentrated *in vacuo*. The crude yellow oil was purified by column chromatography on silica gel (2:1 hexanes/EtOAc) to give a mixture of *Benzyl 3-(6-methyl-1,2,4-triazin-3-ylcarbamoyl)propanoate* and *Benzyl 3-(5-methyl-1,2,4-triazin-3-ylcarbamoyl)propanoate* (3:1) (45 mg, 0.15 mmol, 7%) as an orange solid. R_F (2:1 hexanes/EtOAc) 0.27; δ_H (500 MHz, $CDCl_3$): 8.87 (1 H, s, 5-

methyl- H_6), 8.36 (1 H, s, 6-methyl-H_5), 7.38-7.35 (5 H, m, Ar-*H*), 3.22 – 3.18 (2 H, m, 5-methyl-OCH$_2$CH_2OH), 3.12 (2 H, t, $^3J_{H\text{-}H}$ 6.5, 6-methyl-OCH$_2$CH_2OH), 2.86 – 2.81 (2 H, m, OCH_2CH$_2$OH), 2.68 (3H, s, 6-methyl-CH_3), 2.54 (3 H, s, 5-methyl-CH_3).; δ_C (125 MHz, CDCl$_3$): 173.0 (O*C*OCH$_2$), 160.9 (5/6-methyl-Tz-C_5/C_6), 157.4 (Tz-C_3), 149.9 & 145.9 (5/6-methyl-Trz-*C*H$_5$/*C*H$_6$), 135.8 (Ar-C_1), 128.6, 128.3 & 128.2 (Ar-$C_{2\text{-}6}$), 66.7 (Ar*C*H$_2$), 32.6 & 32.4 (OCH$_2$*C*H$_2$), 28.9 (O*C*H$_2$), 21.8 (5-methyl-Tz-*C*H$_3$), 18.9 (6-methyl-Tz-*C*H$_3$); ν_{max} (solid)/cm^{-1}: 3430 (NH stretch), 1709 (CO); *m/z* (ES): Found *M(+H)* 301.1294, $C_{15}H_{17}N_4O_3$ requires 301.1295; HPLC (5-95% A): retention time 1.88 min, 84%.

Fmoc-Ser-OMe

(9H-Fluoren-9-yl)methyl (S)-1-(methoxycarbonyl)-2-hydroxyethylcarbamate

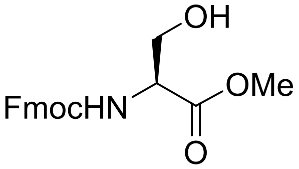

Sodium bicarbonate (6.7 g, 60 mmol, 2.5 eq) was added to a vigorously stirred solution of L-serine methyl ester hydrochloride **10** (5 g, 32 mmol, 1 eq) in H_2O (~60 ml) at 0°C,[29] the turbid solution immediately became clear. Fmoc *N*-hydroxysuccinimide ester (10.8 g, 32 mmol, 1 eq), dissolved in H_2O (~60 ml) was added dropwise and a colourless precipitate formed. The solution was allowed to warm to room temperature and stirred for 4 hours, at which time TLC showed complete consumption of both reagents. The reaction was quenched with H_2O (1 × 50 ml) and extracted with EtOAc (3 × 60 ml). The combined organics were washed with H_2O (2 × 50 ml) and brine (2 × 50 ml); dried ($MgSO_4$) and concentrated on the cold finger. The crude colourless solid was recrystalised from EtOAc/hexanes, cooled to -20 °C, and the resultant crystals collected at the pump, before being washed with ice-cold hexanes (2 × 50 ml) to yield *Fmoc-Ser-Ome* (9.66g, 28 mmol, 88%) as large, colourless crystals. m.p. 128.2-130.6 °C; $[\alpha]_D^{27}$ +4.35 (*c* = 0.12, CHCl$_3$); R_F (3:1 hexanes/EtOAc) 0.17; δ_H (500 MHz, CDCl$_3$): 7.70 (2H, d, $^3J_{H\text{-}H}$ 7.5, Fmoc-H_4), 7.57-7.50 (2H, m, Fmoc-H_1), 7.34 (2H, t, $^3J_{H\text{-}H}$ 7.5, Fmoc-H_3), 7.25 (2H, t, $^3J_{H\text{-}H}$ 7.5, Fmoc-H_2), 5.66-5.59 (1H, m, N*H*), 4.45-4.27 (3H, m, Fmoc-CHCH_2 & H_α), 4.16 (1 H, t, $^3J_{H\text{-}H}$ 6.8, Fmoc-C*H*CH$_2$), 3.94 (1 H, d, $^2J_{H\text{-}H}$ 8.9, H_β), 3.86 (1 H, d, $^2J_{H\text{-}H}$ 10.2, H_β), 3.73 (3 H, s, OCH_3); δ_C (125 MHz, CDCl$_3$): 171.1 (*C*OOCH$_3$), 156.2 (O*C*ONH), 143.7 (Fmoc-C_{4a}), 141.4 & 141.3 (Fmoc-C_{4a}), 127.77 (Fmoc-C_3), 127.1 (Fmoc-C_2), 125.1 (Fmoc-C_1), 120.0 (Fmoc-C_4), 67.2 (Fmoc-CH*C*H$_2$), 63.3 (C_β), 56.03 (C_α), 52.8 (O*C*H$_3$), 47.2 (Fmoc-*C*HCH$_2$); ν_{max} (solid)/cm^{-1}: 3470 & 2431 (NH stretch), 3314 (OH) 1749 (CO); *m/z* (ES): Found: *M(+H)* 342.1339, $C_{19}H_{20}NO_5$ requires 342.1336; HPLC (5-95% B): retention time 2.94 min, 100%.

Fmoc-Ser(OTs)-OMe
(9H-Fluoren-9-yl)methyl (S)-1-(methoxycarbonyl)-2-(*p*-toluenesulfonate)ester

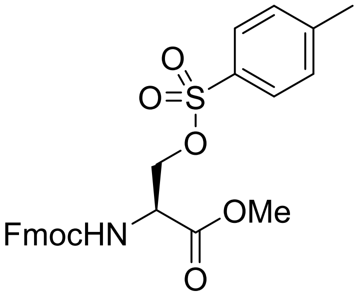

p-Toluenesulfonyl chloride (16.4 g, 86 mmol, 2 eq) was added to a solution of Fmoc-Ser-OMe (14.7 g, 43 mmol, 1 eq) in pyridine (~40 ml) at 0°C.[29] The colourless solution immediately turned pale orange. The reaction mixture was allowed to warm to room temperature and stirred for 16 hours. The resultant clear red solution was quenched with H_2O (1 × 40 ml) and extracted with EtOAc (3 × 60 ml). The combined organics were washed with 1M citric acid solution (3 × 50 ml), saturated $NaHCO_3$ (2 × 50 ml), brine (1 × 40 ml), 1M HCl solution (3 × 50 ml), saturated $NaHCO_3$ (2 × 50 ml) and brine (2 × 40 ml). The organic layer was dried ($MgSO_4$) and concentrated on the cold finger. The colourless solid was recrystalised from 1:1 EtOH/Petroleum ether and collected at the pump. The resultant pale yellow crystals were triturated in ice-cold petroleum ether for 30 minutes and collected at the pump to yield *Fmoc-Ser-OMe* (13.38 g, 27 mmol, 63%) as colourless crystals. m.p. 119.7-120.2 °C; $[\alpha]_D^{27}$ +3.25 (c = 0.14, CH_2Cl_2); R_F (3:1 hexanes/EtOAc) 0.29; δ_H (500 MHz, $CDCl_3$): 7.74 – 7.64 (4 H, m, Fmoc-H_4 & OTs-H_2), 7.56 – 7.49 (2 H, m, Fmoc-H_1), 7.34 (2 H, t, $^3J_{H\text{-}H}$ 7.3, Fmoc-H_3), 7.29-7.23 (2 H, m, Fmoc-H_2), 7.21 (2 H, d, $^3J_{H\text{-}H}$ 8.2, OTs-H_3), 5.55 (1 H, d, $^3J_{H\text{-}H}$ 7.9, NH), 4.53 – 4.48 (1 H, m, H_α), 4.39 (1 H, d, $^2J_{H\text{-}H}$ 7.0, H_β), 4.35 (1H, d, $^2J_{H\text{-}H}$ 7.5, H_β), 4.32 – 4.15 (2 H, m, CHCH_2), 4.12 (1 H, t, $^3J_{H\text{-}H}$ 7.2, CHCH$_2$), 3.67 (3 H, s, OCH_3), 2.29 (3 H, s, OTsCH_3). δ_C (125 MHz, $CDCl_3$): 168.7 (COOCH$_3$) 155.6 (OCONH$_2$), 145.2 (OTs-C_4) 143.6 (Fmoc-C_{4a}) 141.3 (Fmoc-C_{1a}) 132.3 (OTs-C_2), 130.0 (OTs-C_3), 128.0 & 127.8 (Fmoc-C_3), 127.2 (Fmoc-C_2), 125.2, &125.1 (Fmoc-C_1), 120.0 (Fmoc-C_4), 69.0 (C_β), 67.5 (CHCH$_2$), 53.4 (C_α), 53.1 (OCH$_3$), 47.02 (CHCH$_2$), 21.58 (OTs-CH$_3$); ν_{max} (solid)/cm^{-1}: 3428 & 3338 (NH stretch), 1749 (CO); *m/z* (ES): Found: *M(+H)* 496.1428, $C_{26}H_{26}NO_7S$ requires 496.1424; HPLC (5-95% B): retention time 4.10 min, 61%.

Fmoc-IodoAla-OMe 11
(9H-Fluoren-9-yl)methyl (R)-1-(methoxycarbonyl)-2-iodoethylcarbamate

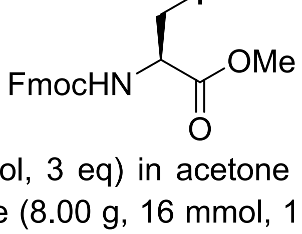

Sodium iodide (7.20 g, 48 mmol, 3 eq) in acetone (80 ml) was added to a stirred solution of Fmoc-Ser(OTs)-Ome (8.00 g, 16 mmol, 1 eq) in acetone (160 ml).[29] The colourless solution turned yellow. After stirring for 16 hours at room temperature the

mixture was filtered at the pump and concentrated *in vacuo* to obtain a yellow oil. The oil was dissolved in DCM (40 ml) and washed with water (3 × 50 ml), saturated sodium thiosulfate solution (2 × 50 ml) and brine (2 × 40 ml). The organic layer was dried ($MgSO_4$), concentrated and purified by column chromatography on silica gel (2:1 DCM/hexanes) to leave a colourless viscous oil. The oil was triturated in an ice-cold solution of 1:1 hexane/EtOH and collected at the pump to yield *Fmoc-IodoAla-OMe* (3.74 g, 8.2 mmol, 52%) as a flocculent, colourless solid. m.p. 148.8-149.4°C; $[\alpha]_D^{27}$ + 4.85 (*c* = 0.11, CH_2Cl_2); R_F (2:1 DCM/hexanes) 0.21; δ_H (500 MHz, $CDCl_3$): 7.78 (2 H, d, $^3J_{H-H}$ 7.5, Fmoc-H_4), 7.63 (2 H, d, $^3J_{H-H}$ 7.4, Fmoc-H_1), 7.42 (2 H, t, $^3J_{H-H}$ 7.5, Fmoc-H_3), 7.34 (2 H, tdd, $^3J_{H-H}$ 7.5, $^4J_{H-H}$ 3.0, $^5J_{H-H}$ 1.1, Fmoc-H_2), 5.69 (1 H, d, *J* 7.3, N*H*), 4.60 (1 H, dt, $^3J_{H-H}$ 7.6, $^3J_{H-H}$ 3.8, H_α), 4.48 – 4.36 (2 H, m, Fmoc-CHCH_2), 4.26 (1 H, t, $^3J_{H-H}$ 7.2, Fmoc-C*H*CH_2), 3.84 (3 H, s, OCH_3), 3.61 (1 H, d, $^3J_{H-H}$ 3.8, H_β); δ_C (125 MHz, $CDCl_3$); 169.7 (*C*$OOCH_3$), 155.4 (O*C*ONH), 143.8 & 143.7 (Fmoc-C_{4a}), 141.3 (Fmoc-C_{1a}), 127.8 (Fmoc-C_3), 127.1 (Fmoc-C_2), 125.2 & 125.1 (Fmoc-C_1), 120.05 (Fmoc-C_4), 67.4 (CH*C*H_2), 54.1 (C_α) 53.2 & 53.1 (O*C*H_3), 45.2 (*C*HCH_2) 7.4 (C_β); ν_{max} (solid)/cm^{-1}: 3743 & 3338 (NH stretch), 1749 (CO); *m/z* (ES) Found: *M(+H)*, 452.0349, $C_{19}H_{19}INO_4$ requires 452.0353; HPLC (5-95% B): retention time 3.91 min, 72%.

FITC-Gaba-TrzAla-CONH$_2$ 14

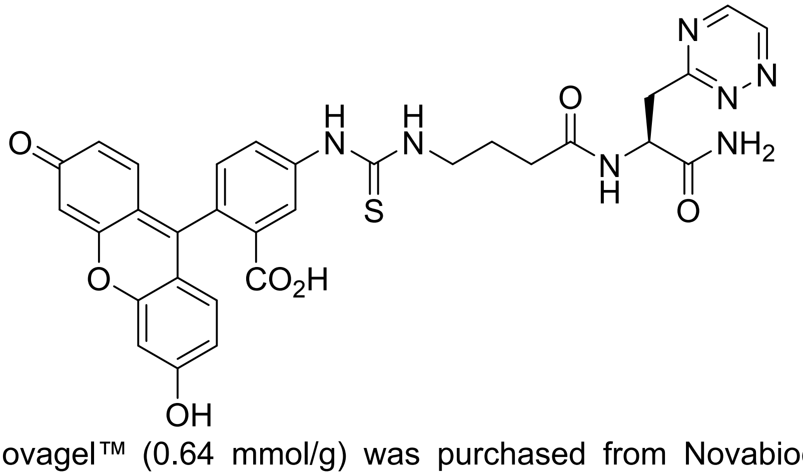

Rink Amide Novagel™ (0.64 mmol/g) was purchased from Novabiochem, Fmoc-Gaba-OH[1] and fluorescein isothiocyanate isomer 1 (FITC) were purchased from Sigma Aldrich.

FITC-Ga-TrzA-CONH$_2$ was synthesised manually using standard solid phase synthesis protocol: Rink Amide Novagel™ (0.032 mmol) was swollen in DMF (2-3 ml-the same volume was used for the wash steps) for 30 min with agitation (Stuart Rotator SB2). The solution was then removed by vacuum filtration and the first coupling mixture added. Coupling reactions of Fmoc-TrzAla-OH **13** (3 eq. with respect to resin, 0.096 mmol, 37 mg) and Fmoc-Gaba-OH (5 eq. with respect to resin, 0.16 mmol, 65 mg) were carried out using 5 eq. HCTU and 10 eq. DIPEA in

[1] Gaba = γ-aminobutyric acid

DMF (1 mL) for 60 minutes with agitation. After each coupling reaction the solution was removed by filtration and the resin was washed with DMF (3 × 2 min), 20% piperidine in DMF (*v/v*, 5 × 2 min) and DMF (5 × 2 min). The peptide N-termini was appended to fluorescein by mixing the resin with a solution containing FITC (5 eq, 0.16 mmol, 60 mg) and DIPEA (5 eq, 0.8 mmol, 165 μl) for 16 h in the dark with agitation. The solution was removed by filtration and the resin was washed with DMF (3 × 2 min), DCM (3 × 2 min) and MeOH (3 × 2 min) and dried for 2 h *in vacuo*. The peptide was cleaved from the resin by mixing with a cleavage cocktail (2ml) consisting of TFA (95%), H_2O (2.5%) and TIS (2.5%). The cleavage cocktail was applied to the resin and mixed for 2 h, then precipitated into ice-cold diethyl ether (30 ml) and the precipitate collected by centrifugation (4,000 × *g*, 10 minutes). The ethereal supernatant was decanted, the peptide pellet resuspended in ice-cold diethyl ether and centrifuged again. This was repeated 2 more times before residual ether was removed under a stream of nitrogen. The resultant amorphous solid was dissolved in the minimum volume H_2O (with the addition of small portions of Dioxane), frozen and lyophilized to give the crude material as a flocculent orange solid. *FITC-Ga-TrzA-CONH*$_2$ was purified by mass directed HPLC (5-95% MeCN + 0.1% formic acid), the volatiles were removed *in vacuo* and the remaining solution frozen and lyophilized to leave the title compound (7.52 mg, 0.12 mmol, 37%) as a flocculent orange solid.

(Z,1S,8R,9r)-9-(Ethoxymethyl)bicyclo[6.1.0]non-4-ene 17a

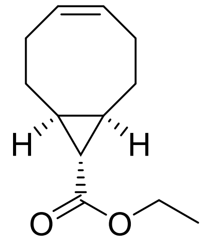

1,5-cyclooctadiene (35 ml, 283 mmol, 8 eq), rhodium (II) acetate (94 mg, 0.22 mmol, 0.01 eq) were added to a flask which was evacuated and charged with nitrogen three times.[36] The reactants were vigorously stirred in dry DCM (20 ml) and ethyl diazoacetate (85% in DCM, 4.4 ml, 35 mmol, 1 eq) was added dropwise via a syringe pump over 12 hours. The resultant orange solution was concentrated *in vacuo*, and purified using flash chromatography on silica eluting with hexanes, and after collection of excess 1,5-cyclooctadiene, 1% Et_2O in hexanes, to *leave (Z,1S,8R,9r)-9-(Ethoxymethyl)bicyclo[6.1.0]non-4-ene* (3.17 g, 46%, 18 mmol) (combined isomers: 6.11 g, 34 mmol, 89%) as a colourless oil. $[\alpha]_D^{27}$ +1.0 (*c* = 0.07, CH_2Cl_2); R_F (1 % Et_2O in hexanes) 0.24; δ_H (500 MHz, $CDCl_3$): 5.68 – 5.60 (2 H, m, H_4), 4.14 – 4.07 (2 H, m, CH_2CH_3), 2.34 – 2.27 (2 H, m, H_3), 2.23 – 2.16 (2 H, m, H_2), 2.13 – 2.05 (2 H, m, $H_{3'}$), 1.59 – 1.54 (2 H, m, H_1), 1.48 (2 H, dddd, $^3J_{H-H}$ 11.0, $^3J_{H-H}$ 8.7, $^3J_{H-H}$ 5.4, $^2J_{H-H}$ 2.9, $H_{2'}$), 1.25 (2 H, 2, $^3J_{H-H}$ 7.2, CH_2CH_3), 1.19 (1 H, t, $^3J_{H-H}$ 4.6, H_{1a}); δ_C (125 MHz, $CDCl_3$): 174.4 (*C*OOEt), 129.9 (C_4), 60.2 (CH_2CH_3), 28.3 (C_2), 27.9 (C_{1a}), 27.7 (C_1), 26.6 (C_3), 14.20 (CH_2CH_3); ν_{max} (solid)/cm^{-1}: 2934 (alkyne), 1095 (ether); HPLC (5-95% B): retention time 3.77 min, 99%.

(Z,1S,8R,9s)-9-(Ethoxymethyl)bicyclo[6.1.0]non-4-ene 17b

(Z,1S,8R,9s)-9-(ethoxymethyl)bicyclo[6.1.0]non-4-ene was synthesised alongside (Z,1S,8R,9r)-9-(ethoxymethyl)bicyclo[6.1.0]non-4-ene and gave the title compound (2.94 g, 16 mmol, 43%) as a colourless oil. $[\alpha]_D^{27}$ +3.2 (*c* = 0.03, CH_2Cl_2); R_F (1 % Et_2O in hexanes) 0.33; δ_H (500 MHz, $CDCl_3$): 5.65 – 5.58 (2 H, m, Fmoc-H_4), 4.12 (2 H, q, $^3J_{H\text{-}H}$ 7.1, CH_2CH_3), 2.55 – 2.46 (2 H, m, Fmoc-H_3), 2.25 – 2.17 (2 H, m, Fmoc-H_2), 2.10 – 2.02 (2 H, m, Fmoc-$H_{3'}$), 1.88 – 1.79 (2 H, m, Fmoc-H_{2a}), 1.81 (1 H, t, $^3J_{H\text{-}H}$ 8.8, Fmoc-H_1), 1.44 – 1.35 (2 H, m, Fmoc-$H_{2'}$), 1.26 (2 H, t, $^3J_{H\text{-}H}$ 7.1, CH_2CH_3). δ_C (125 MHz, $CDCl_3$): 172.31 (CH_2OEt), 129.47 (C_4), 59.72 (CH_2CH_3), 27.09 (C_3), 24.18 (C_{2a}), 22.68 (C_2), 21.27 (C_1), 14.41 (CH_2CH_3); ν_{max} (solid)/cm^{-1}: 2937 (alkyne), 1010 (ether); HPLC (5-95% B): retention time 4.00 min, 100%.

(Z,1S,8R,9r)-Bicyclo[6.1.0]non-4-ene-9-ylmethanol 18

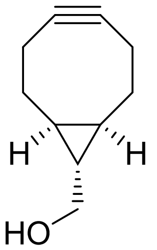

Under a nitrogen atmosphere, a solution of (Z,1S,8R,9r)-9-(ethoxymethyl)bicyclo[6.1.0]non-4-ene **17a** (2.8 g, 14.3 mmol, 1 eq) in DCM (50 ml) was added dropwise to a stirred suspension of lithium aluminium hydride (1.1 g, 28.8 mmol, 2 eq) in DCM (50 ml) at 0°C. The suspension was allowed to warm to room temperature and stirred for 2 hours until TLC showed complete reduction of the ester (3:1 hexanes/EtOAc). The mixture was cooled to 0°C and quenched with sodium sulphate decahydrate (~10 g), filtered through a celite pad, concentrated and azeotroped with toluene to leave a colourless oil. Under a nitrogen atmosphere the reduction product was dissolved in DCM (50 ml) and stirred at 0°C before a solution of bromine (600μl, 13.4 mmol, 1.3 eq) in DCM (9 ml) was added dropwise until a persistent red colour was observed. The reaction was quenched with 10% sodium thiosulphate solution (80 ml), the organics were combined and washed with H_2O (2 × 80 ml), brine (1 × 70 ml), concentrated and azeotroped with toluene. The resultant yellow oil was dissolved in THF (100 ml) under a nitrogen atmosphere and cooled to 0°C before potassium *tert*-butoxide (1M in THF, 43 ml) was added dropwise. The solution was heated to reflux and stirred for 16 hours before being cooled to room temperature, quenched with saturated ammonium chloride solution (100 ml), extracted with DCM (3 × 100 ml), brine (1 × 100 ml), dried ($MgSO_4$) and concentrated to leave an orange oil. The crude residue was purified by column chromatography on silica gel eluting with 1% Et_2O in DCM to leave *(Z,1S,8R,9r)-bicyclo[6.1.0]non-4-ene-9-ylmethanol* (333 mg, 2.21 mmol, 16 %) as a viscous yellow oil. $[\alpha]_D^{27}$ +2.9 (*c* = 0.12, CH_2Cl_2); R_F (1 % Et_2O in hexanes) 0.17; δ_H (500 MHz, $CDCl_3$): 3.56 (2 H, d, $^3J_{H\text{-}H}$ 6.3, CH_2OH), 2.42 (2 H, dd, $^2J_{H\text{-}H}$ 13.3, $^3J_{H\text{-}H}$ 2.7, H_2), 2.33 – 2.25 (2 H, m, H_3), 2.20 – 2.13 (2 H, m, $H_{3'}$), 1.45 – 1.35 (2 H, m, $H_{2'}$), 0.74

– 0.64 (3 H, m. H_{2a+1}). δ_C (125 MHz, $CDCl_3$): 98.8 (C_4), 67.2 (CH_2OH), 33.4 (C_2), 27.3 (C_1), 22.6 (C_{2a}), 21.4 (C_3); ν_{max} (solid)/cm^{-1}: 3350 (OH stretch), 2923 (alkyne); HPLC (5-95% B): retention time 1.85 min, 86%.

(1S,8R,9r)-9-((Benzoyloxy)methyl)bicyclo[6.1.0]non-4-yne 19 (BCN-Bz)

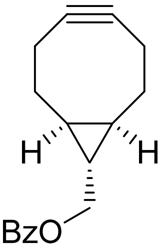

(Z,1S,8R,9r)-bicyclo[6.1.0]non-4-ene-9-ylmethanol **18** (159 mg, 1.06 mmol, 1 eq) and 4-dimethylaminopyridine (8 mg, 0.06 mmol, 0.06 eq) were stirred in DCM (10 ml) under a nitrogen atmosphere and freshly distilled triethylamine (0.45 ml, 1.32 mmol, 3 eq) was added.[37] The solution was cooled to 0°C and benzoyl chloride (0.24 ml, 2.11 mmol, 2 eq) was added dropwise, the colourless solution became amber. The reaction mixture was allowed to warm to room temperature and stirred for 4 hours until TLC showed complete consumption of starting material (4:1 hexanes/EtOAc). The reaction was quenched with saturated ammonium chloride solution (10 ml) and extracted with DCM (3 × 20 ml), the combined organics were washed with brine (2 × 15 ml), dried ($MgSO_4$) and concentrated to leave a brown oil, which was purified by column chromatography on silica (25:1 hexanes/EtOAc) to leave *(Z,1S,8R,9r)-Bicyclo[6.1.0]non-4-ene-9-ylmethanol* (144 mg, 0.59 mmol, 57 %) as a colourless solid. $[\alpha]_D^{27}$ +1.2 (*c* = 0.07, CH_2Cl_2); R_F (25: 1 hexanes/EtOAc) 0.09; δ_H (500 MHz, $CDCl_3$): 8.08 – 8.04 (2 H, m, Bz-H_2), 7.59 – 7.55 (1 H, m, Bz-H_4), 7.45 (2 H, t, $^3J_{H-H}$ 7.8, Bz-H_3), 4.27 (2 H, d, $^3J_{H-H}$ 6.4,OCOCH_2), 2.48 – 2.41 (2 H, m, H_3), 2.36-2.27 (2 H, m, H_2), 2.18 (2 H, dd, $^2J_{H-H}$ 9.4, $^3J_{H-H}$ 5.6, $H_{2'}$), 1.42 (2 H, d, $^2J_{H-H}$ 11.5, $H_{3'}$), 0.90 – 0.80 (3 H, m, H_{2a+1}); δ_C (125 MHz, $CDCl_3$): 132.83 (Bz-C_4), 130.5 (Bz-C_1), 129.6 (Bz-C_2), 128.3 (Bz-C_3), 98.8 (C_4), 69.1 (CH_2OH), 33.3 (C_2), 23.6 (C_1), 23.1 (C_{2a}), 21.4 (C_3); ν_{max} (solid)/cm^{-1}: 2227 (alkyne), 1263 (ether); HPLC (5-95% B): retention time 3.93 min, 100%.

General procedure for HPLC assay

Fmoc-TrzAla-OMe **13** (9H-Fluoren-9-yl)methyl (S)-1-(methoxycarbonyl)-2-(1,2,4-triazin-3-yl)ethylcarbamate) (0.73 mg, 1.80 μmol, 1 eq) was dissolved in 900 μl of the required solvent mix (either 100 % MeCN or 10% H_20). BCN-Bz **19** ((1S,8R,9r)-9-((Benzoyloxy)methyl)bicyclo[6.1.0]non-4-yne) was dissolved in 900 μl of the required solvent mix ((0.46 mg, 1.8 μmol), (0.92 mg, 3.6 μmol), (1.83 mg, 7.20 μmol) or (3.66 mg (14.3 μmol) with respect to a final concentration (in 1.8 ml) of 1 mM, 2 mM, 4 mM or 8 mM respectively. The two solutions were combined, mixed thoroughly and capped before being immediately incubated at 37 °C in the auto loader of an Agilent technologies 1290 HPLC, fitted with an ACE Ultracore 2.5 Super C18 column (50 × 2.1 mm).
Each 2 μL sample was injected and analysed using a linear gradient (5% H_2O, in MeCN to 95% H_2O) over 5 min. Typically 4 reactions of varying concentration in respect to BCN-Bz were assayed in parallel. After every analysis, 2 μl of MeCN was injected onto the column and the column washed using the same linear gradient (5% H_2O, 0.5% TFA in MeCN to 95% H_2O, 0.5% TFA) over 9 min. HPLC analysis was carried out over a 20 hour period. Concentration of the cycloaddition product was measured against a previously measured concentration dilution series of the product. A typical HPLC trace (figure S3) and calibration graph (Figure S2) are shown below.

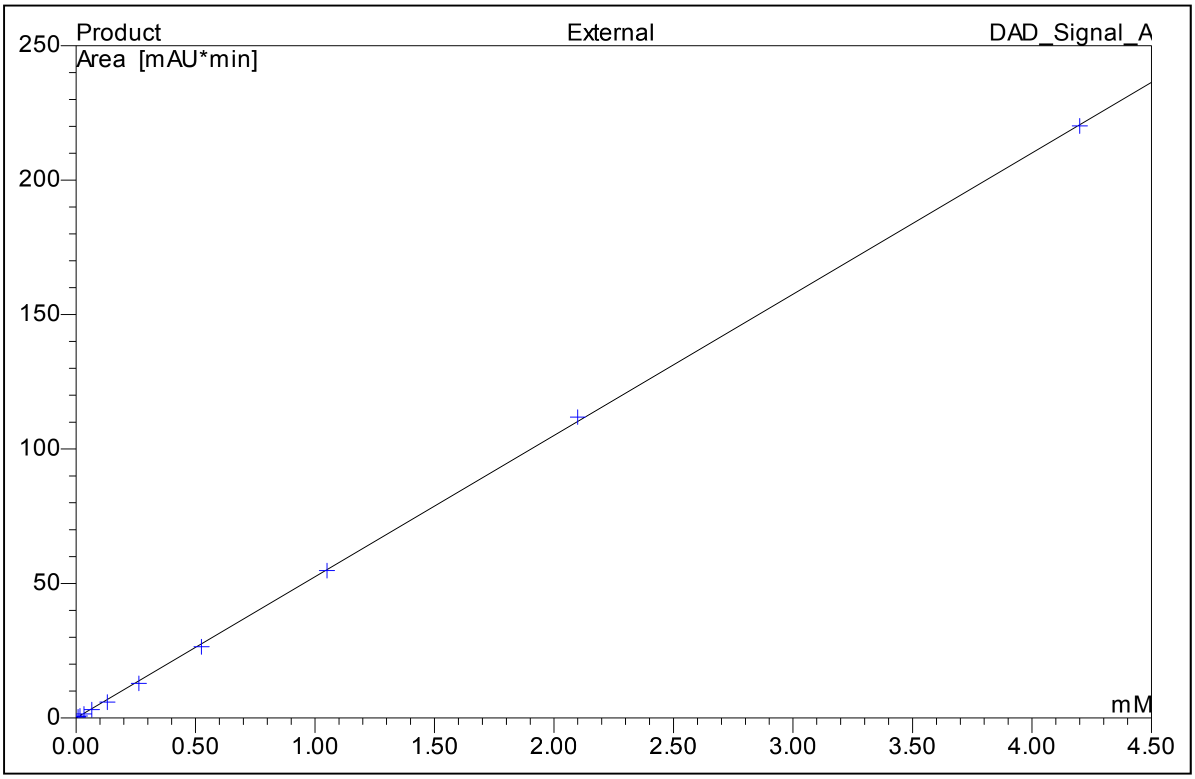

Figure S2 Calibration curve for determination of cycloaddition product formation by HPLC.

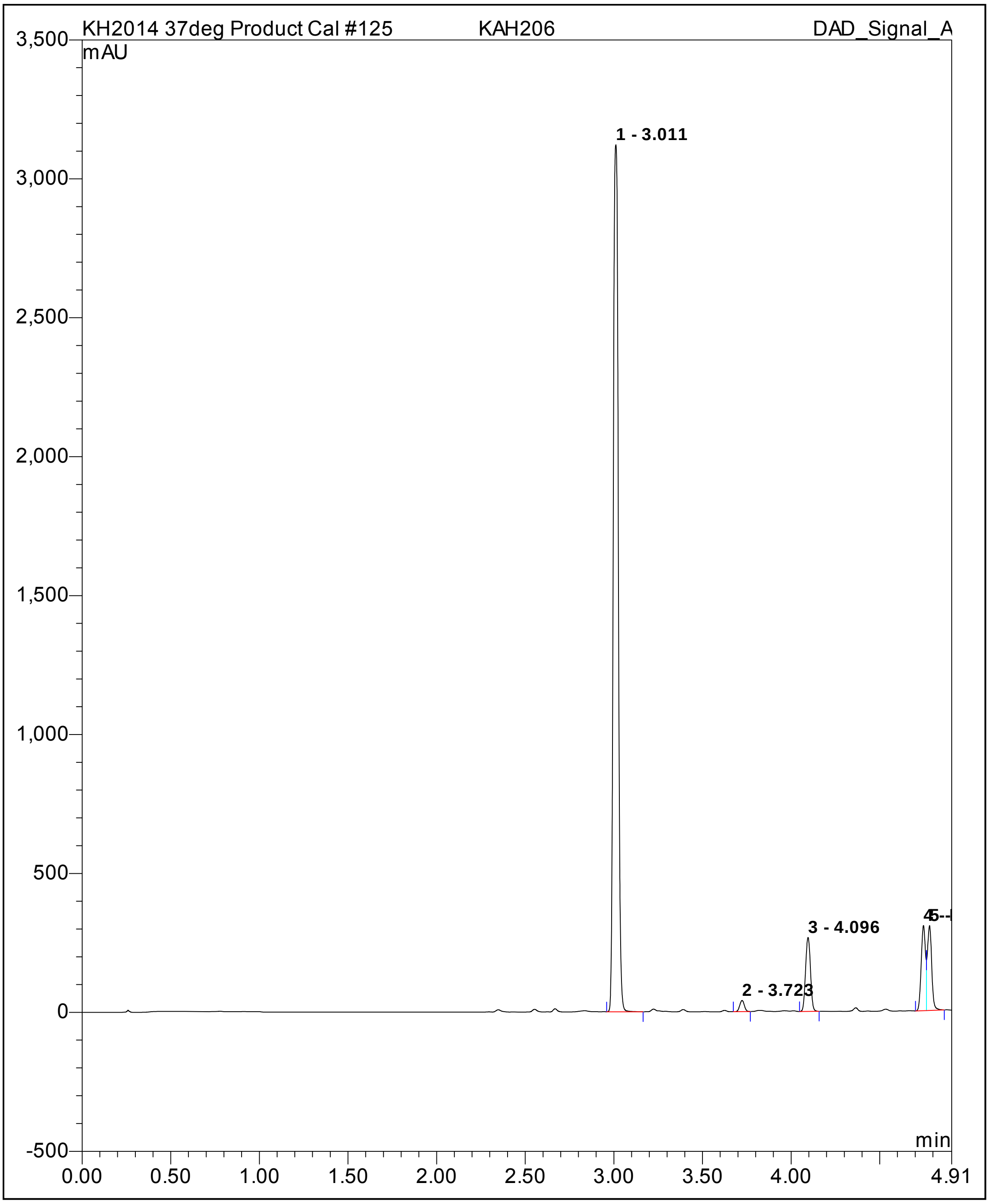

Figure S3 Representative chromatogram from cycloaddition rate determination. 1. Fmoc-TrzAla-OMe, 3- BCN-Bz, 4/5 20a and 20b

NMR spectra, High-resolution mass spectra and HPLC chromatograms

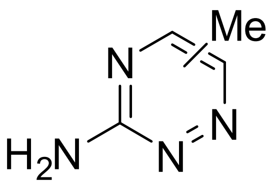
Me
N
N
N
H_2N

5b

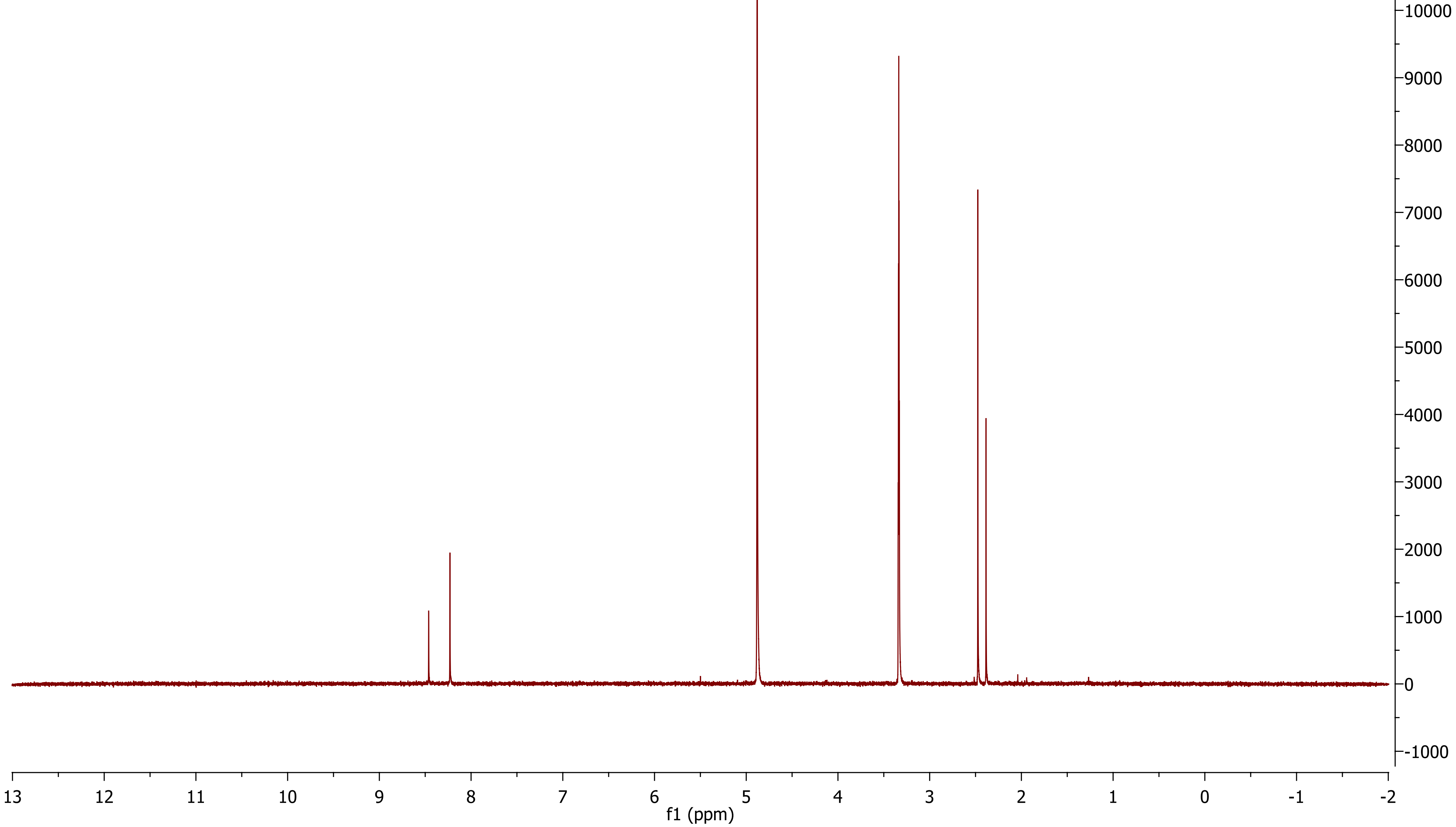
12000
11000
10000
9000
8000
7000
6000
5000
4000
3000
2000
1000
0
-1000
13 12 11 10 9 8 7 6 5 4 3 2 1 0 -1 -2
f1 (ppm)

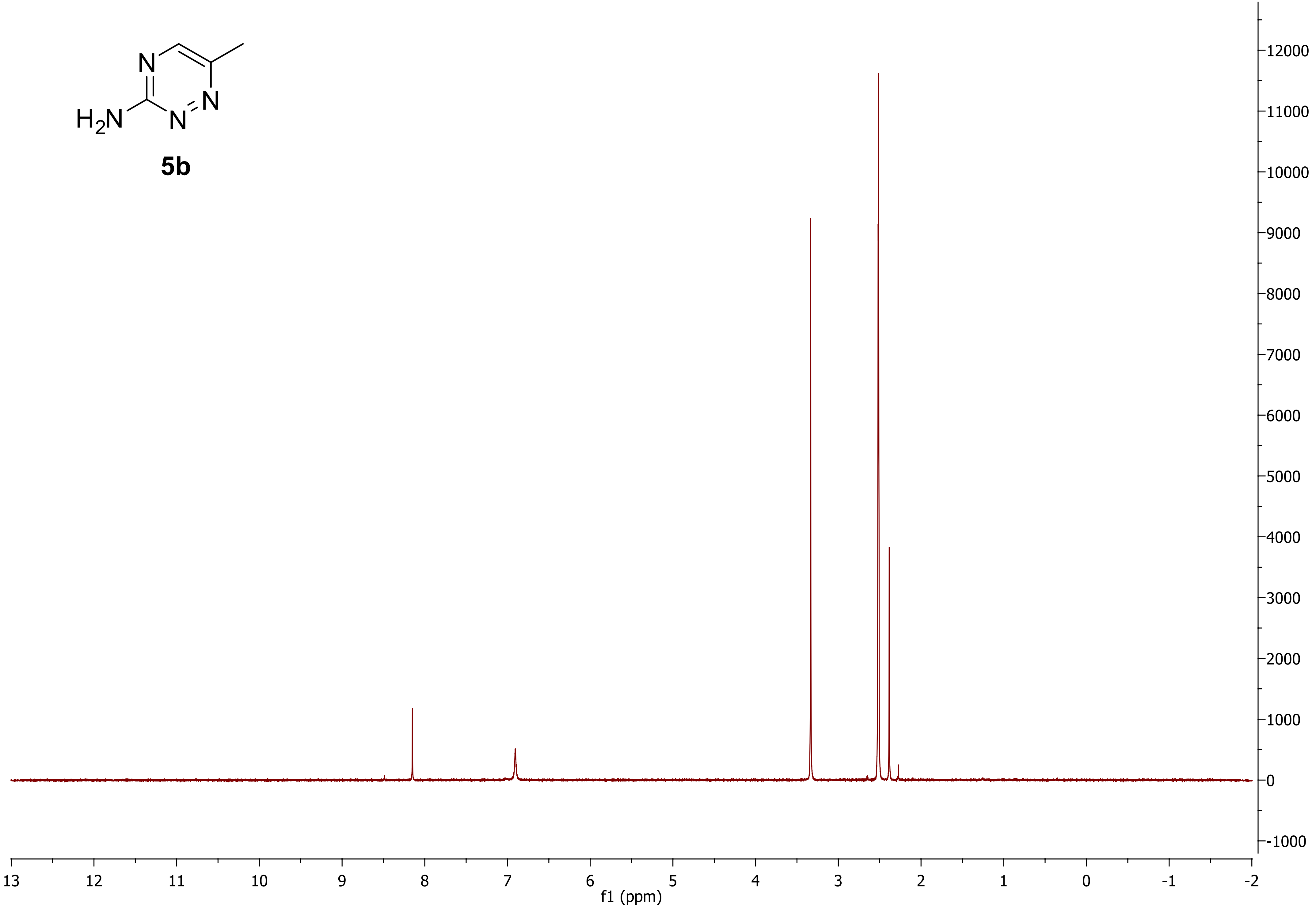
N
N
N
H2N
5b
12000
11000
10000
9000
8000
7000
6000
5000
4000
3000
2000
1000
0
-1000
13
12
11
10
9
8
7
6
5
4
3
2
1
0
-1
-2
f1 (ppm)

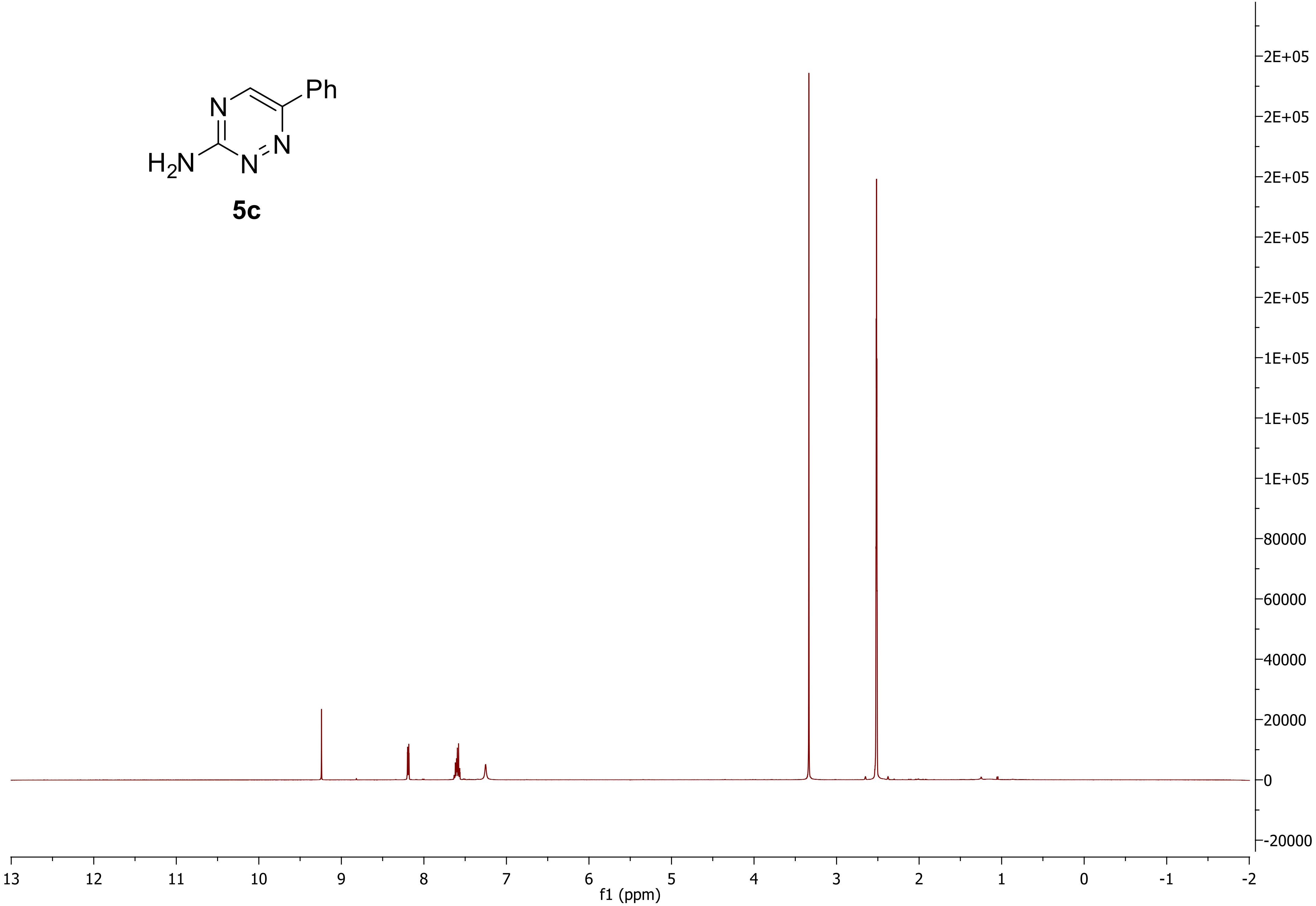

Ph
N
N
H_2N
N
5c
2E+05
2E+05
2E+05
2E+05
2E+05
1E+05
1E+05
1E+05
80000
60000
40000
20000
0
-20000
13
12
11
10
9
8
7
6
5
4
3
2
1
0
-1
-2
f1 (ppm)

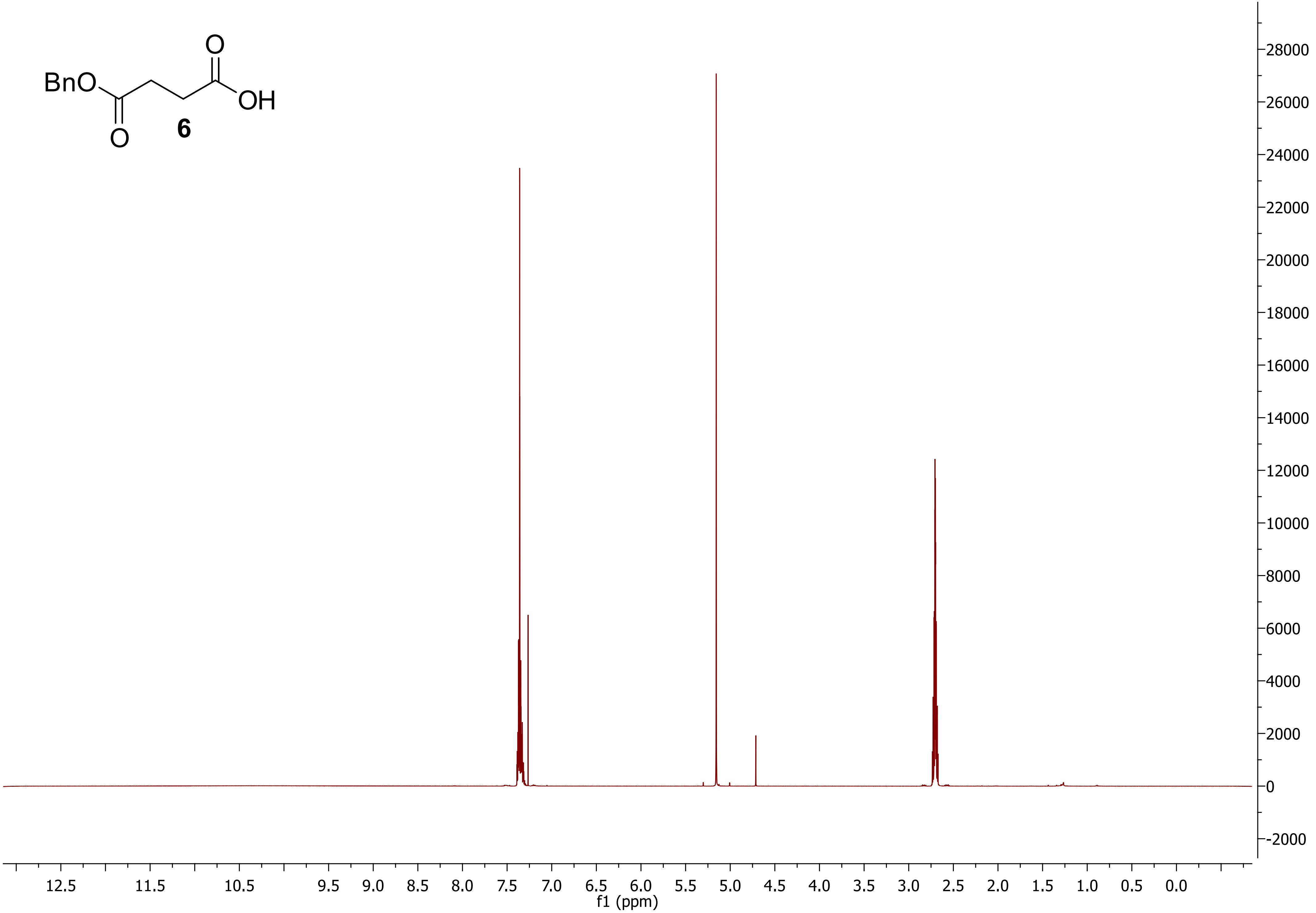
BnO
OH
6
f1 (ppm)
12.5
11.5
10.5
9.5
9.0
8.5
8.0
7.5
7.0
6.5
6.0
5.5
5.0
4.5
4.0
3.5
3.0
2.5
2.0
1.5
1.0
0.5
0.0
28000
26000
24000
22000
20000
18000
16000
14000
12000
10000
8000
6000
4000
2000
0
-2000

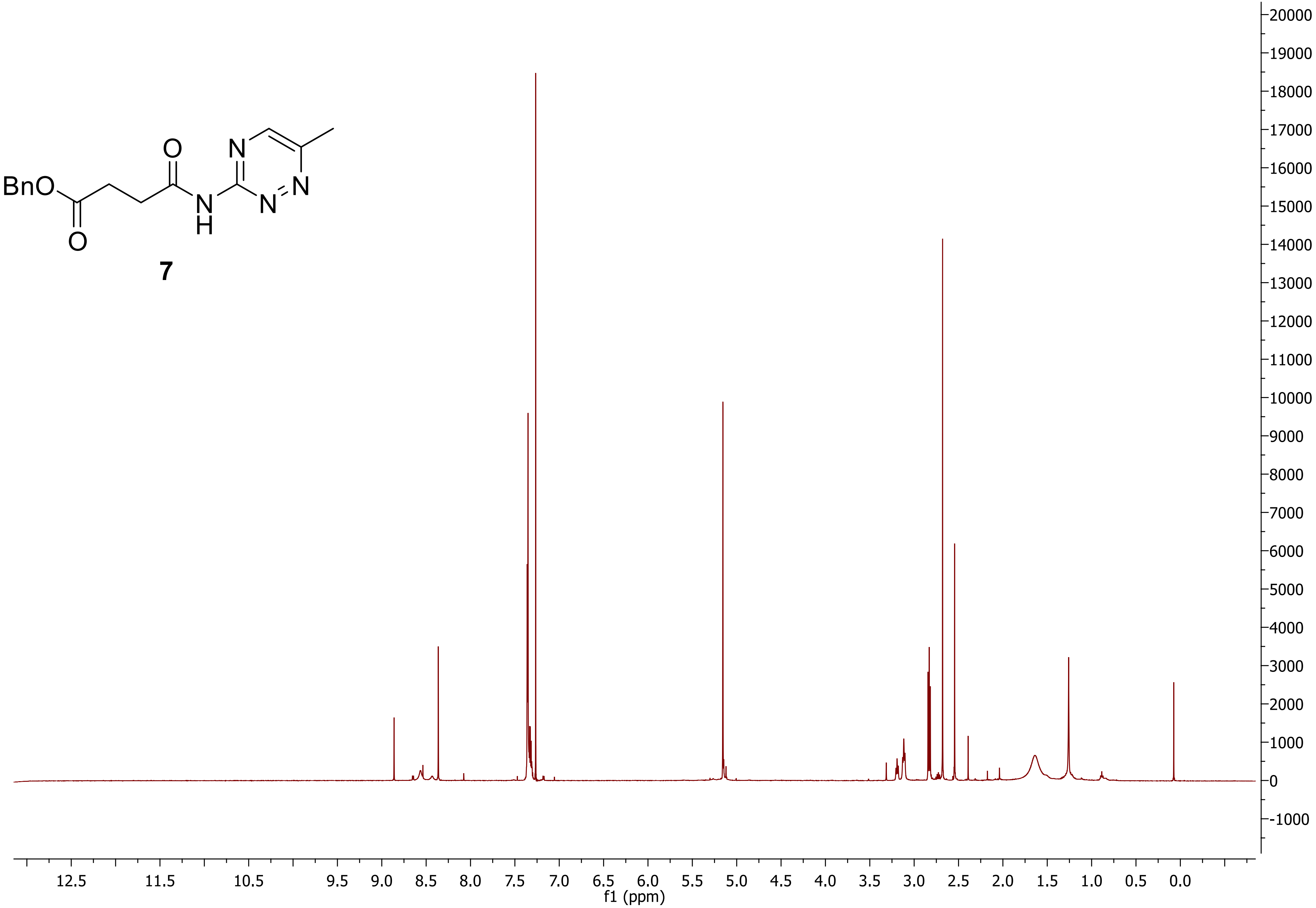

7
20000
19000
18000
17000
16000
15000
14000
13000
12000
11000
10000
9000
8000
7000
6000
5000
4000
3000
2000
1000
0
-1000
12.5 11.5 10.5 9.5 9.0 8.5 8.0 7.5 7.0 6.5 6.0 5.5 5.0 4.5 4.0 3.5 3.0 2.5 2.0 1.5 1.0 0.5 0.0
f1 (ppm)

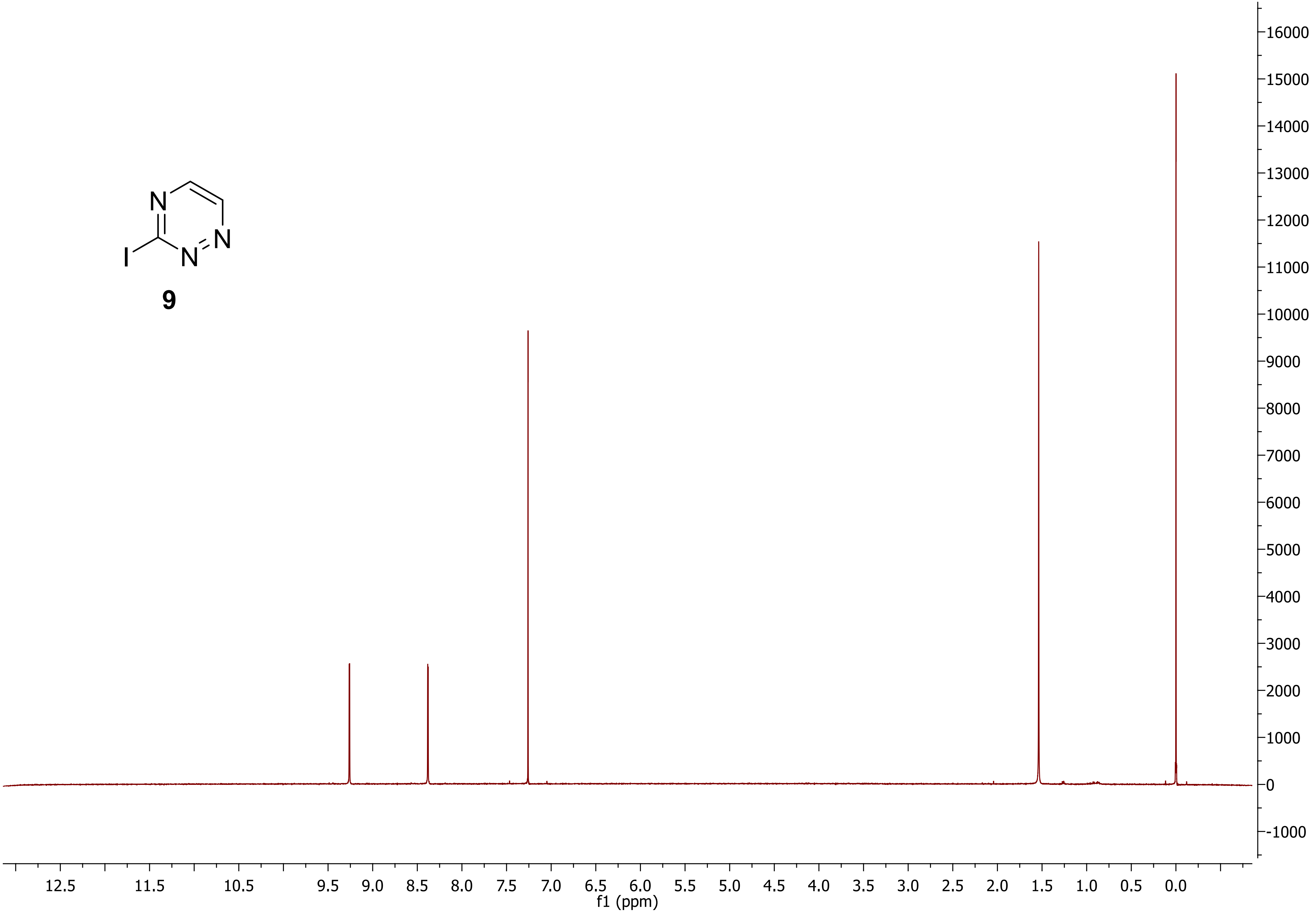

9

16000
15000
14000
13000
12000
11000
10000
9000
8000
7000
6000
5000
4000
3000
2000
1000
0
-1000
12.5 11.5 10.5 9.5 9.0 8.5 8.0 7.5 7.0 6.5 6.0 5.5 5.0 4.5 4.0 3.5 3.0 2.5 2.0 1.5 1.0 0.5 0.0
f1 (ppm)

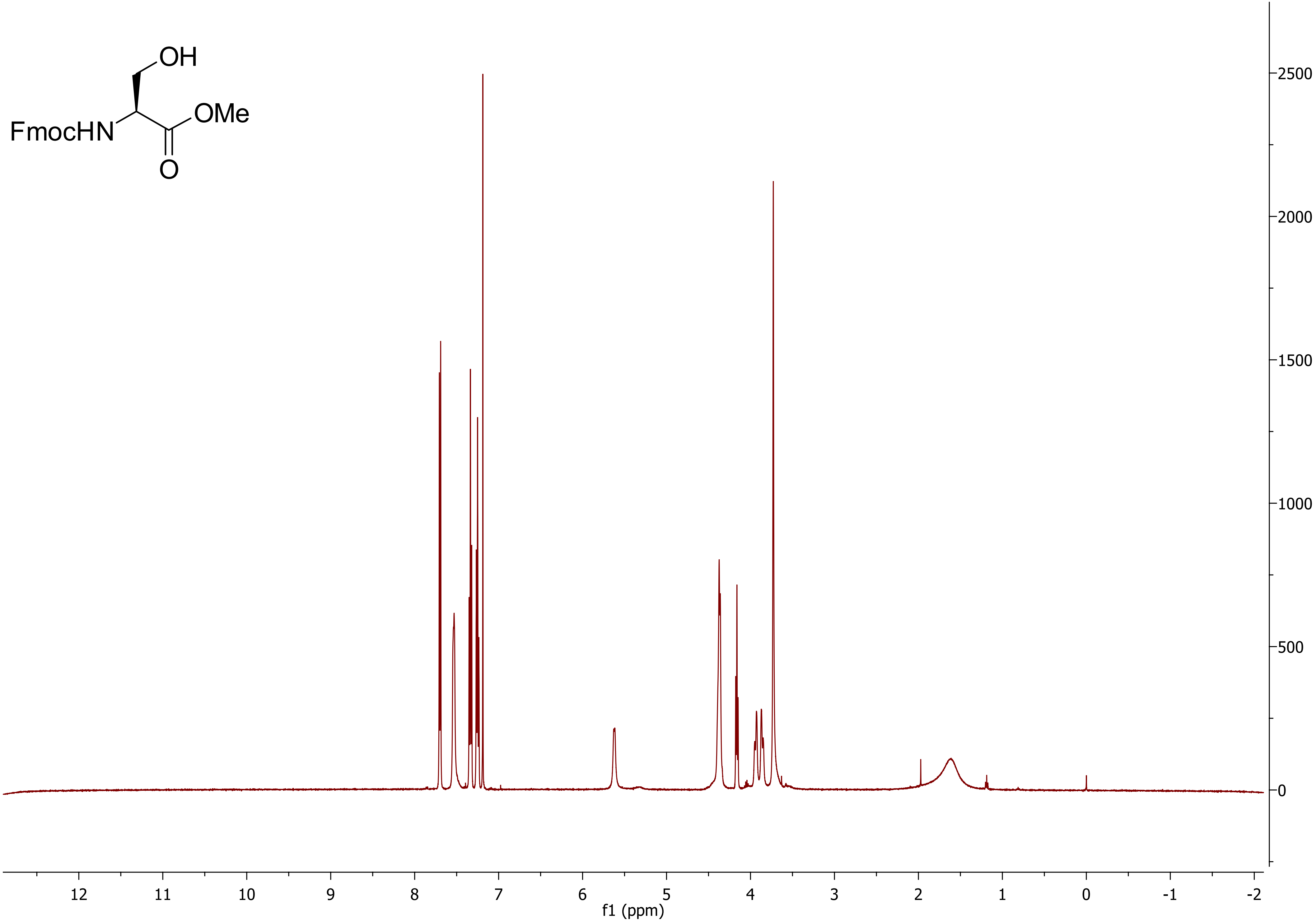

OH
OMe
FmocHN
O
2500
2000
1500
1000
500
0
12
11
10
9
8
7
6
5
4
3
2
1
0
-1
-2
f1 (ppm)

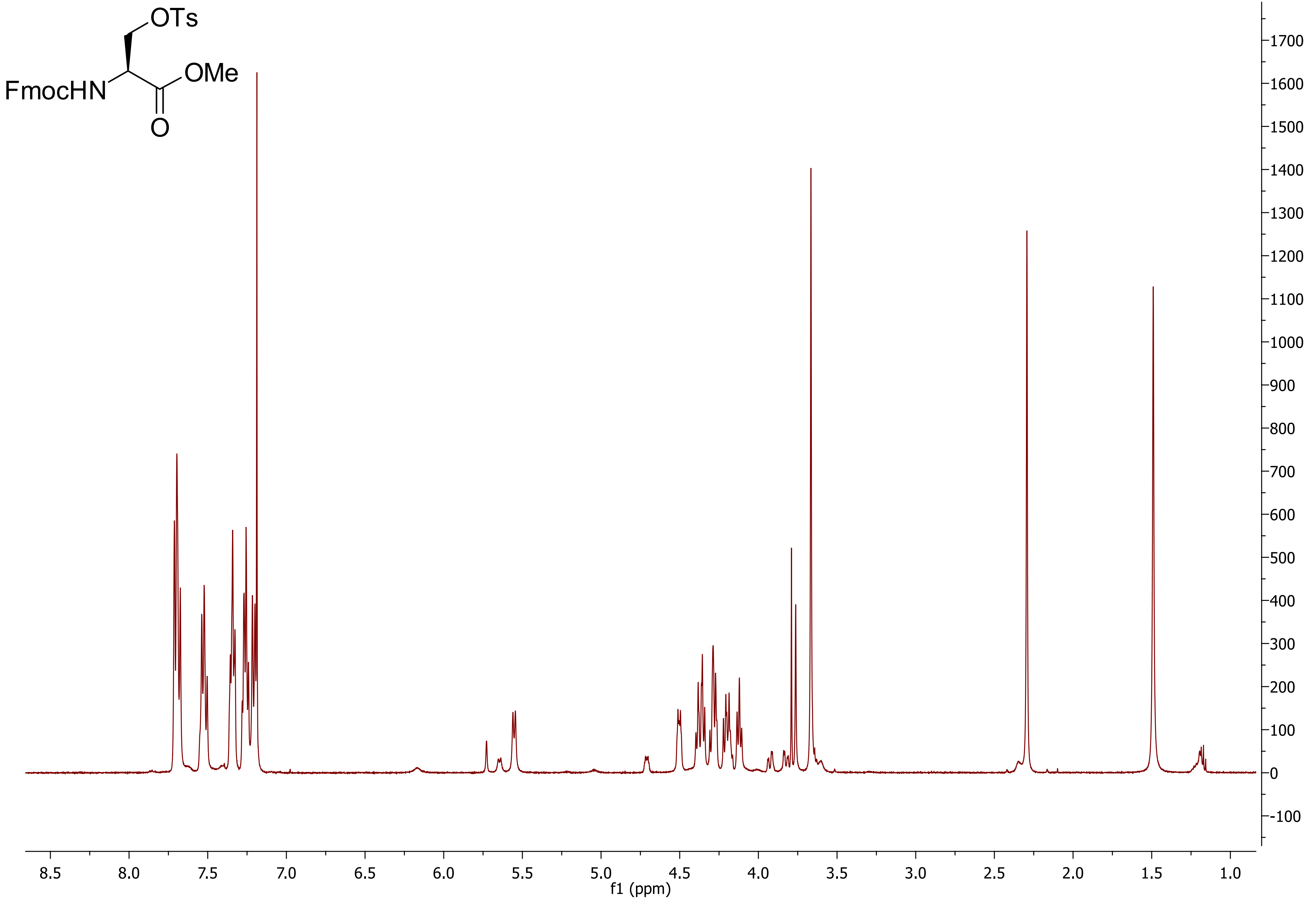
OTs
OMe
FmocHN
O
f1 (ppm)
8.5
8.0
7.5
7.0
6.5
6.0
5.5
5.0
4.5
4.0
3.5
3.0
2.5
2.0
1.5
1.0
1700
1600
1500
1400
1300
1200
1100
1000
900
800
700
600
500
400
300
200
100
0
-100

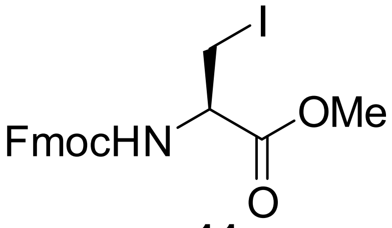

I
OMe
FmocHN
O

11

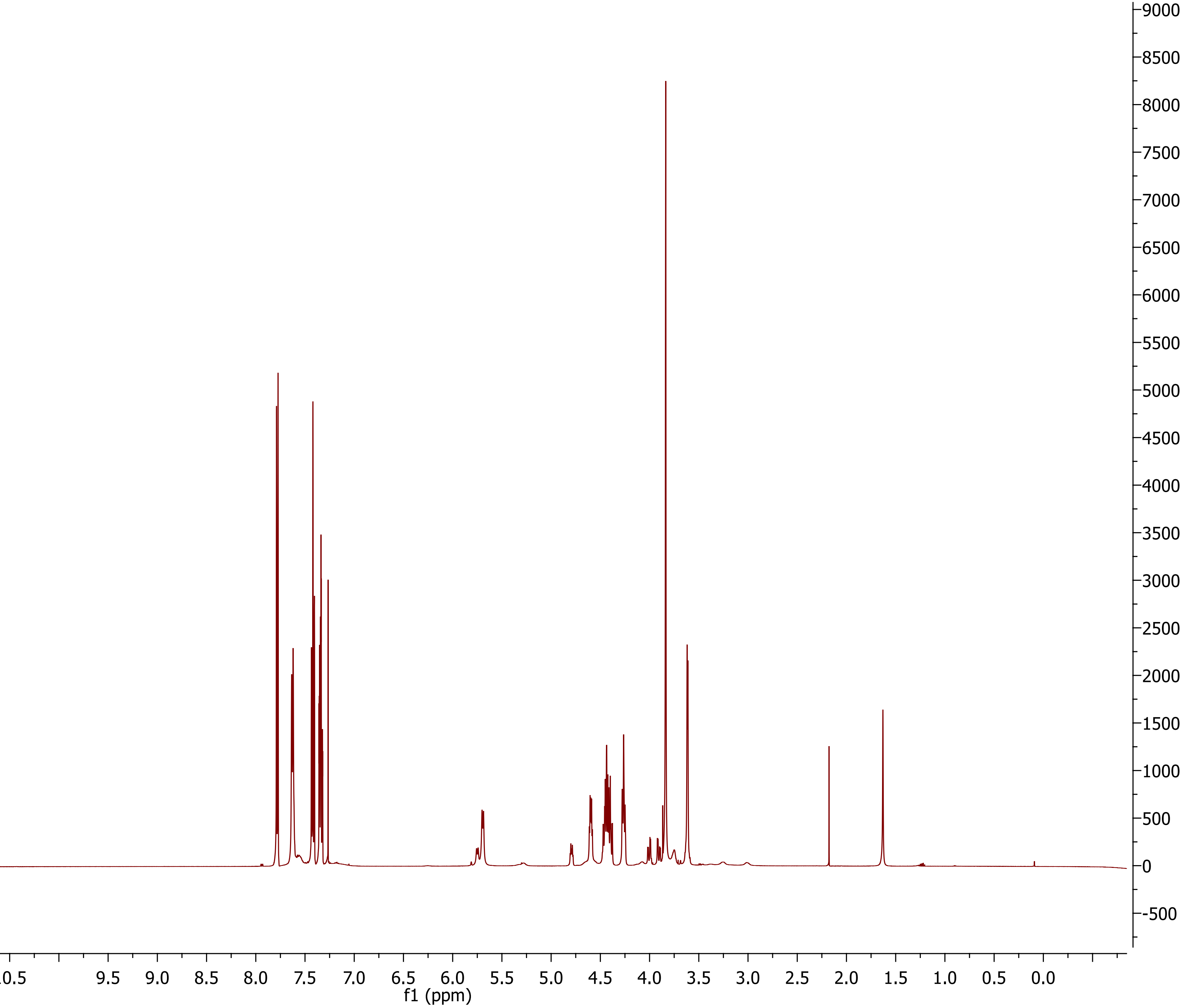

9000
8500
8000
7500
7000
6500
6000
5500
5000
4500
4000
3500
3000
2500
2000
1500
1000
500
0
-500
12.5
11.5
10.5
9.5
9.0
8.5
8.0
7.5
7.0
6.5
6.0
5.5
5.0
4.5
4.0
3.5
3.0
2.5
2.0
1.5
1.0
0.5
0.0
f1 (ppm)

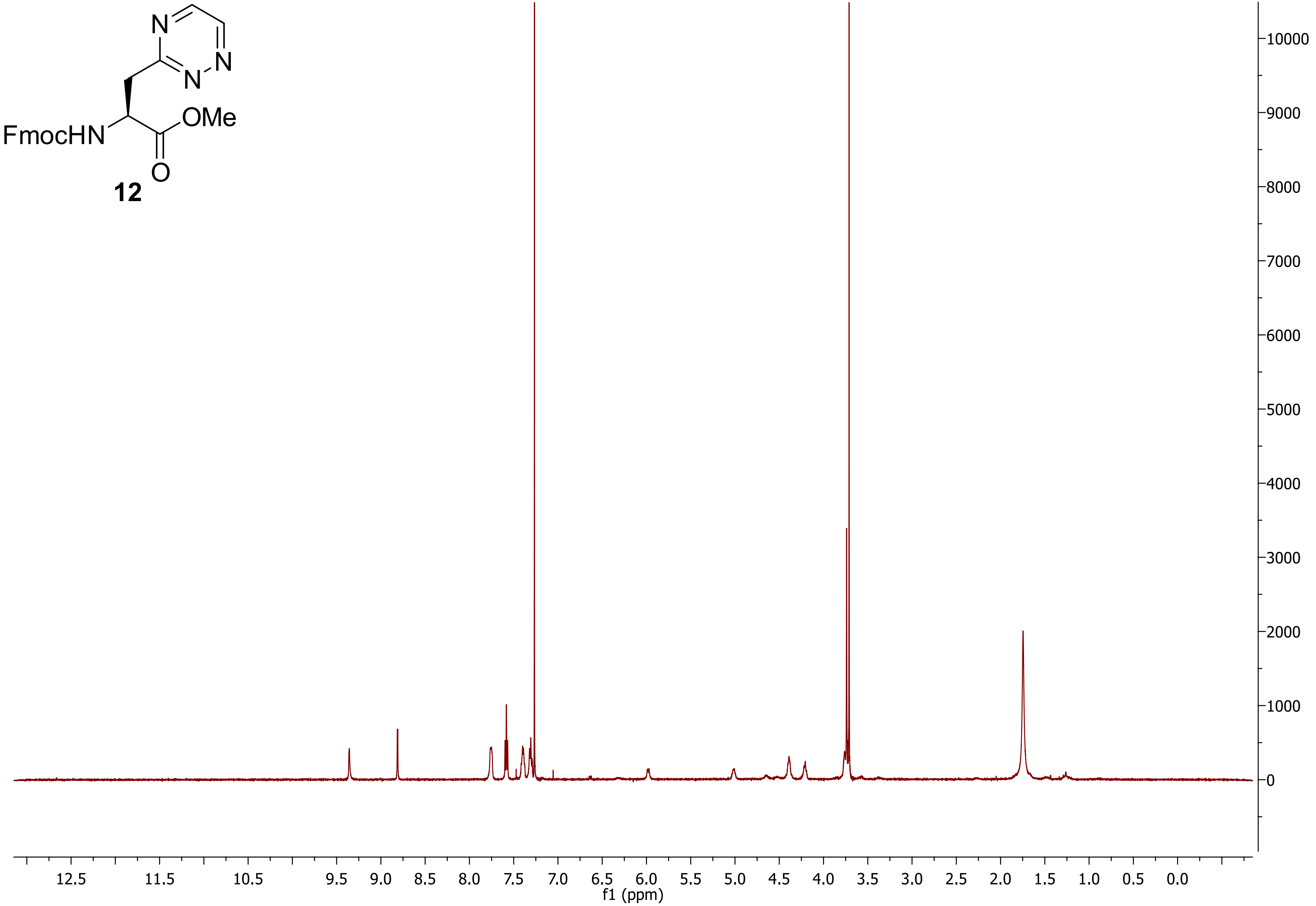
FmocHN
OMe
O
N
N
N
12
10000
9000
8000
7000
6000
5000
4000
3000
2000
1000
0
12.5
11.5
10.5
9.5
9.0
8.5
8.0
7.5
7.0
6.5
6.0
5.5
5.0
4.5
4.0
3.5
3.0
2.5
2.0
1.5
1.0
0.5
0.0
f1 (ppm)

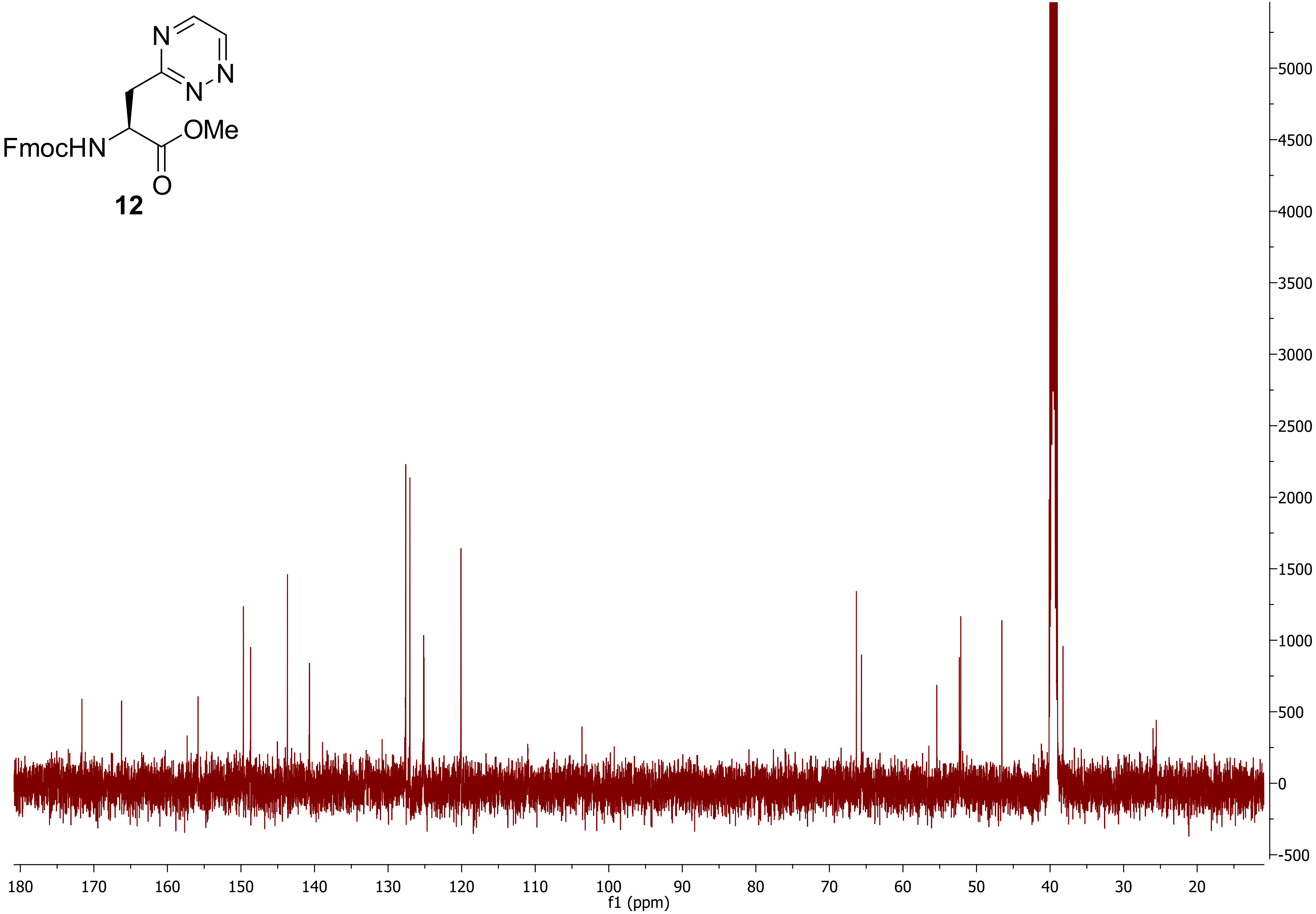
N
N
N
OMe
FmocHN
O
12
180 170 160 150 140 130 120 110 100 90 80 70 60 50 40 30 20
f1 (ppm)
5000
4500
4000
3500
3000
2500
2000
1500
1000
500
0
-500

School of Chemistry Mass Spectrometry Service

**SampleID**	kah176 HRMS			**Submitter**	Katherine Horner		
**Sample Description**							
**Analysis Name**	kah176 HRMS_107361_GE1_01_6623.d			**Supervisor**	Stuart Warriner		
**Method**	3a_AccMass_Loop_Positive.m			**Acquisition Date**	28/07/2014 19:01:26		
**Instrument**	maXis impact	**Source Type**	ESI	**Ion Polarity**	Positive		
**Scan Begin**	50 m/z	**Scan End**	1500 m/z				

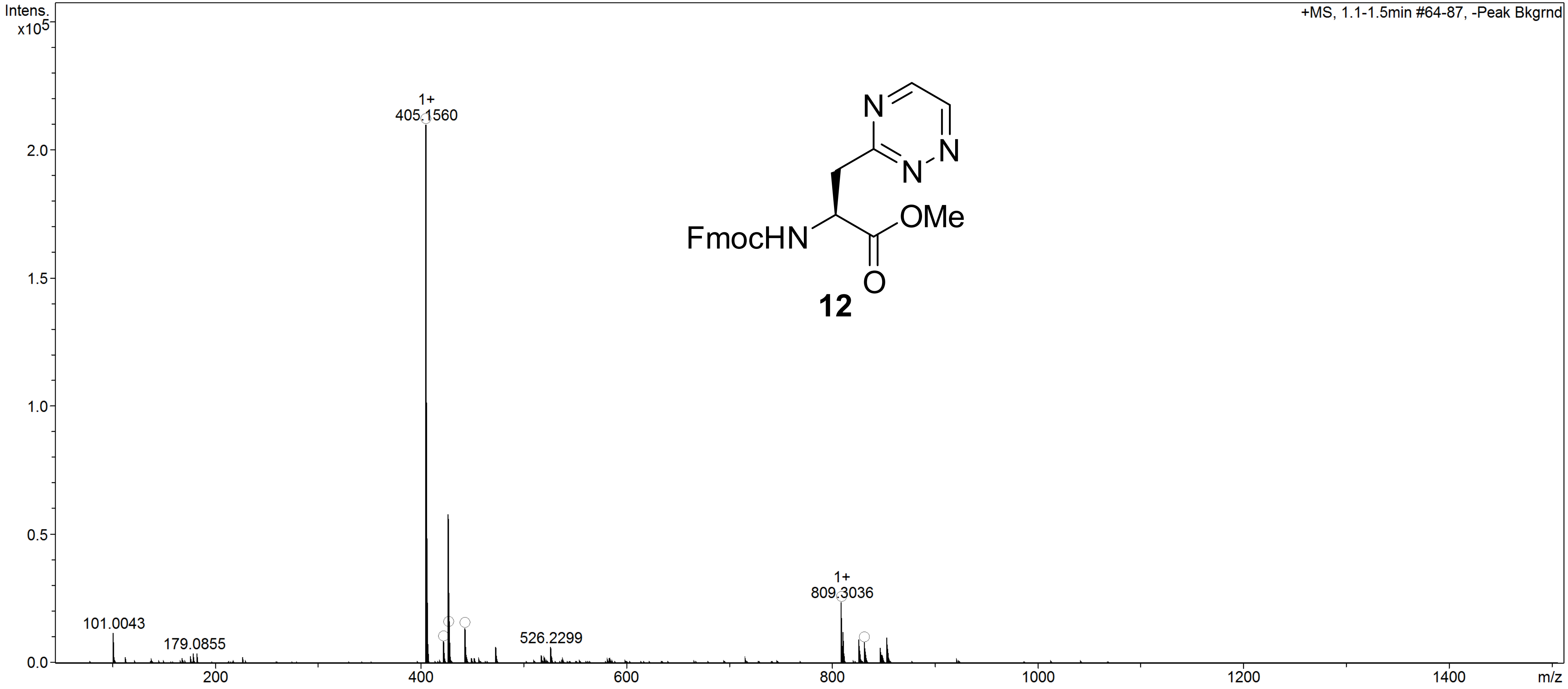

15 176-6 0.12Mm

Sample Name:	**176-6 0.12Mm**	*Injection Volume:*	**1.0**
Vial Number:	**P1:A6**	*Channel:*	**DAD_Signal**
Sample Type:	**standard**	*Wavelength:*	**n.a.**
Control Program:	**Rapid5-95 Ascentis Express C18**	*Bandwidth:*	**n.a.**
Quantif. Method:	**MartinH**	*Dilution Factor:*	**1.0000**
Recording Time:	**30/06/2014 14:22**	*Sample Weight:*	**1.0000**
Run Time (min):	**4.92**	*Sample Amount:*	**1.0000**

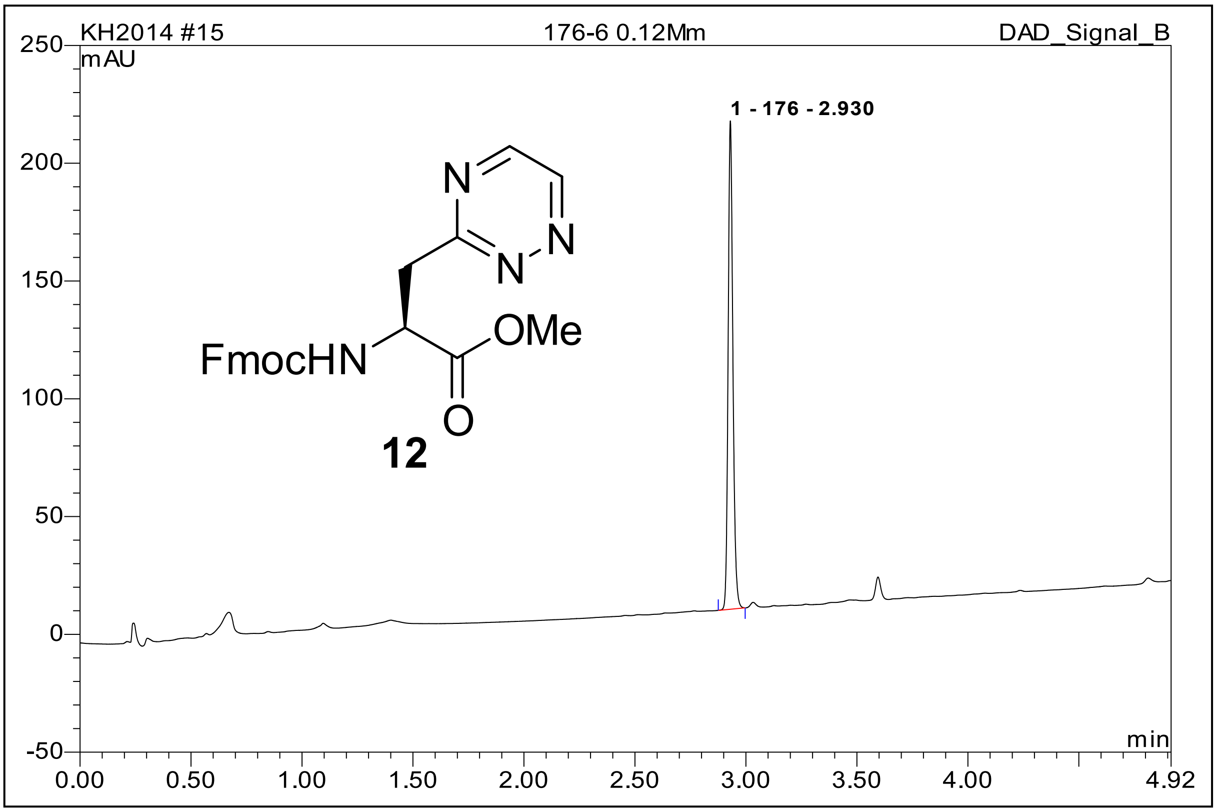

No.	Ret.Time min	Peak Name	Height mAU	Area mAU*min	Rel.Area %	Amount Mm	Type
1	2.93	176	207.314	5.170	100.00	0.172	BMB
**Total:**			207.314	5.170	100.00	0.172	

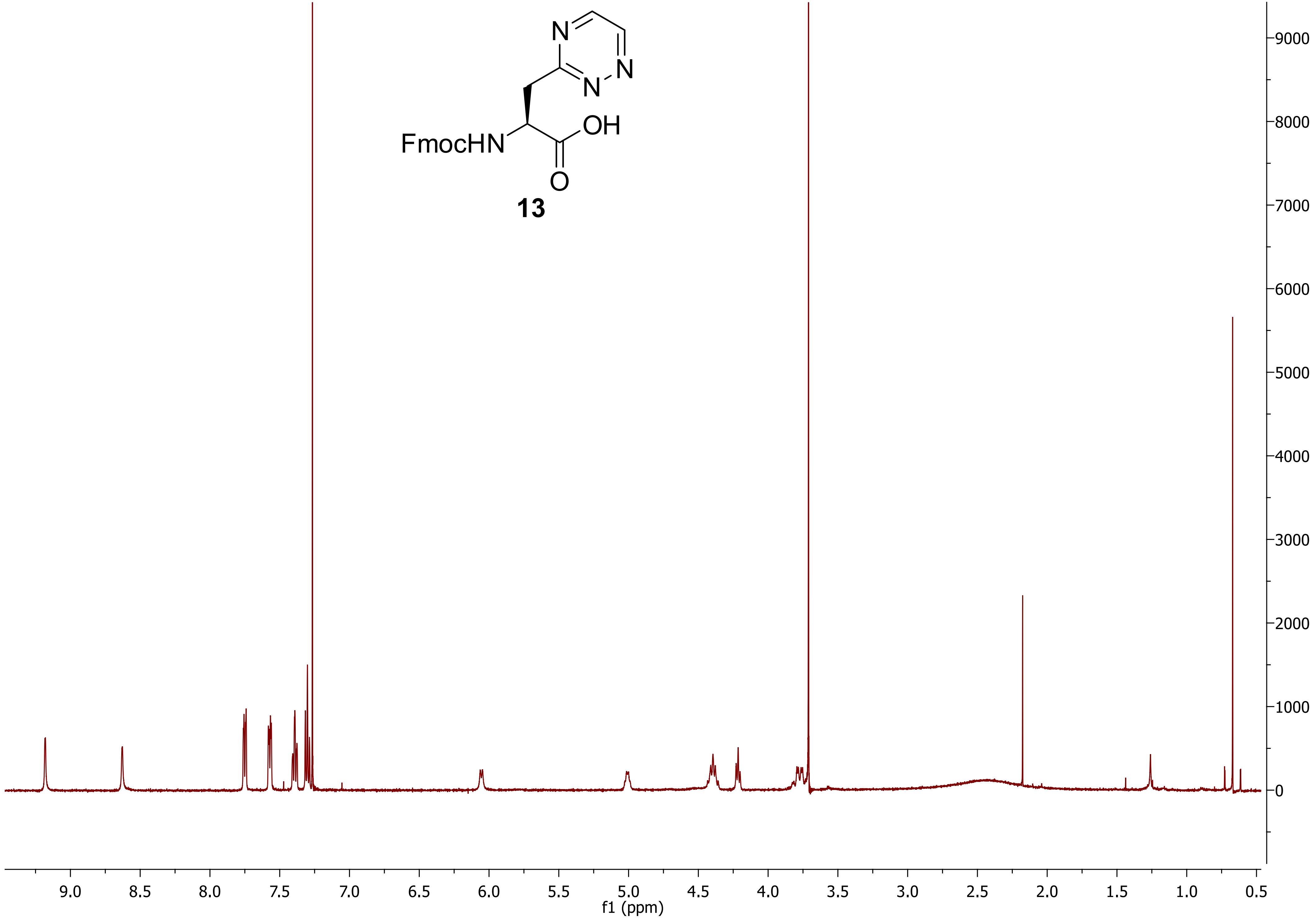
FmocHN
OH
O
N
N
N
9000
8000
7000
6000
5000
4000
3000
2000
1000
0
9.0
8.5
8.0
7.5
7.0
6.5
6.0
5.5
5.0
4.5
4.0
3.5
3.0
2.5
2.0
1.5
1.0
0.5
f1 (ppm)

13

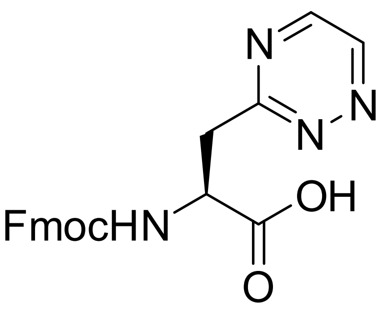

N
N
N
OH
FmocHN
O

13

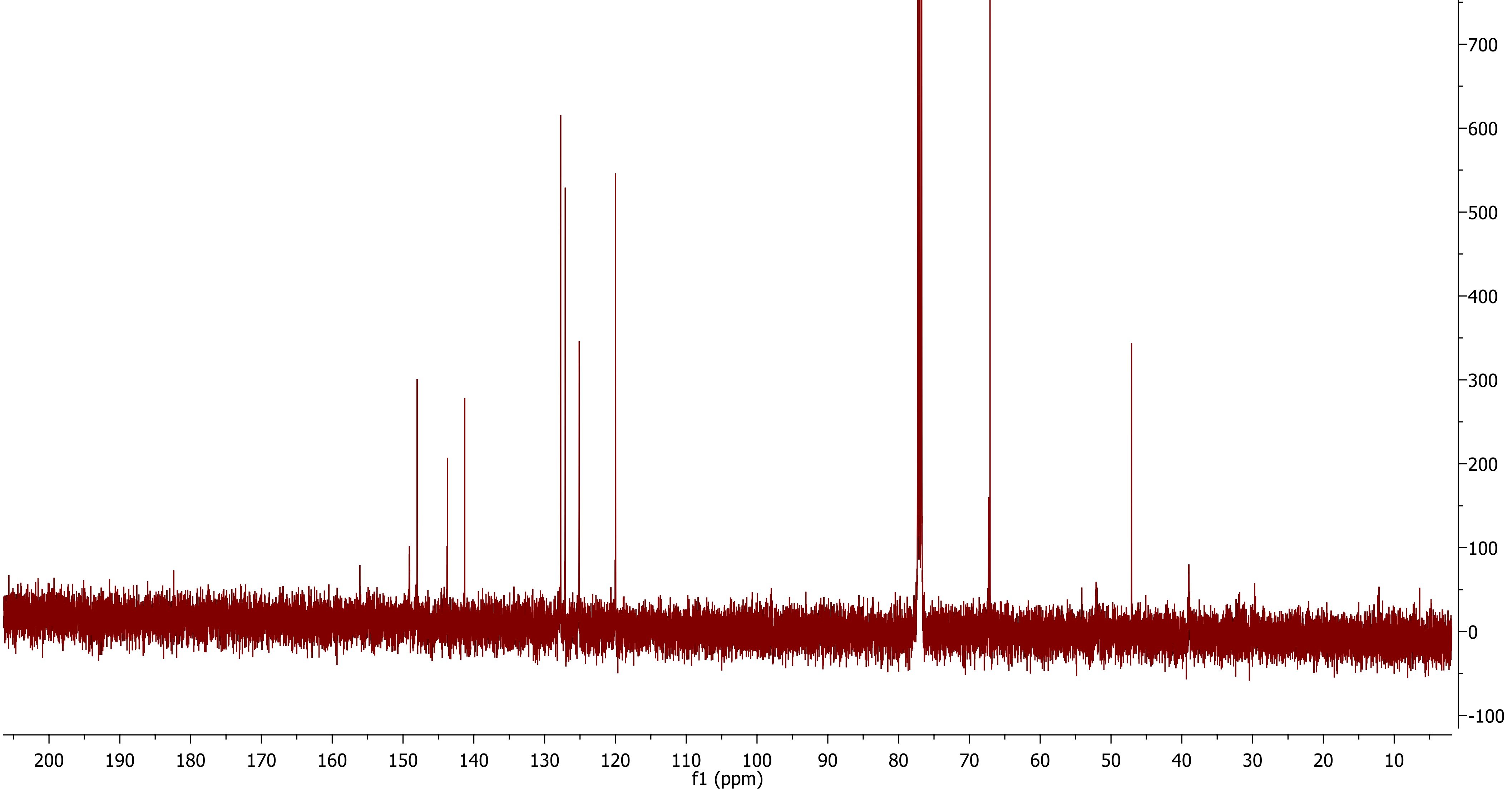

f1 (ppm)
200 190 180 170 160 150 140 130 120 110 100 90 80 70 60 50 40 30 20 10
1000 900 800 700 600 500 400 300 200 100 0 -100

School of Chemistry Mass Spectrometry Service

**SampleID**	KAH192	**Submitter**	Katherine Horner
**Sample Description**			
**Analysis Name**	KAH192_107362_GE2_01_6624.d	**Supervisor**	Stuart Warriner
**Method**	3a_AccMass_Loop_Positive.m	**Acquisition Date**	28/07/2014 19:05:15
**Instrument**	maXis impact **Source Type** ESI **Ion Polarity** Positive	**Scan Begin** 50 m/z	**Scan End** 1500 m/z

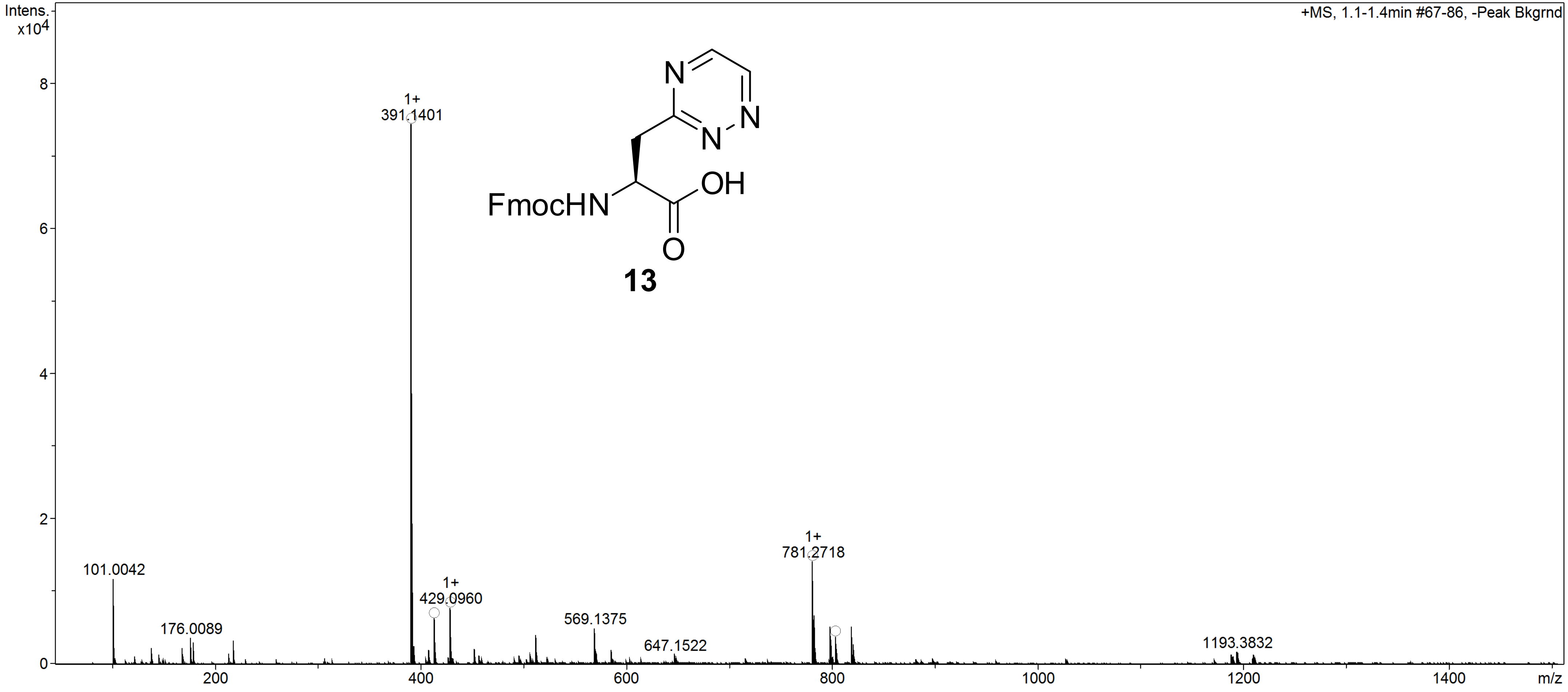

4 KAH192

Sample Name:	**KAH192**	*Injection Volume:*	**1.0**
Vial Number:	**P1:A4**	*Channel:*	**DAD_Signal**
Sample Type:	**unknown**	*Wavelength:*	**n.a.**
Control Program:	**Rapid5-95 Ace UltraCore C18**	*Bandwidth:*	**n.a.**
Quantif. Method:	**MartinH**	*Dilution Factor:*	**1.0000**
Recording Time:	**22/07/2014 08:41**	*Sample Weight:*	**1.0000**
Run Time (min):	**4.91**	*Sample Amount:*	**1.0000**

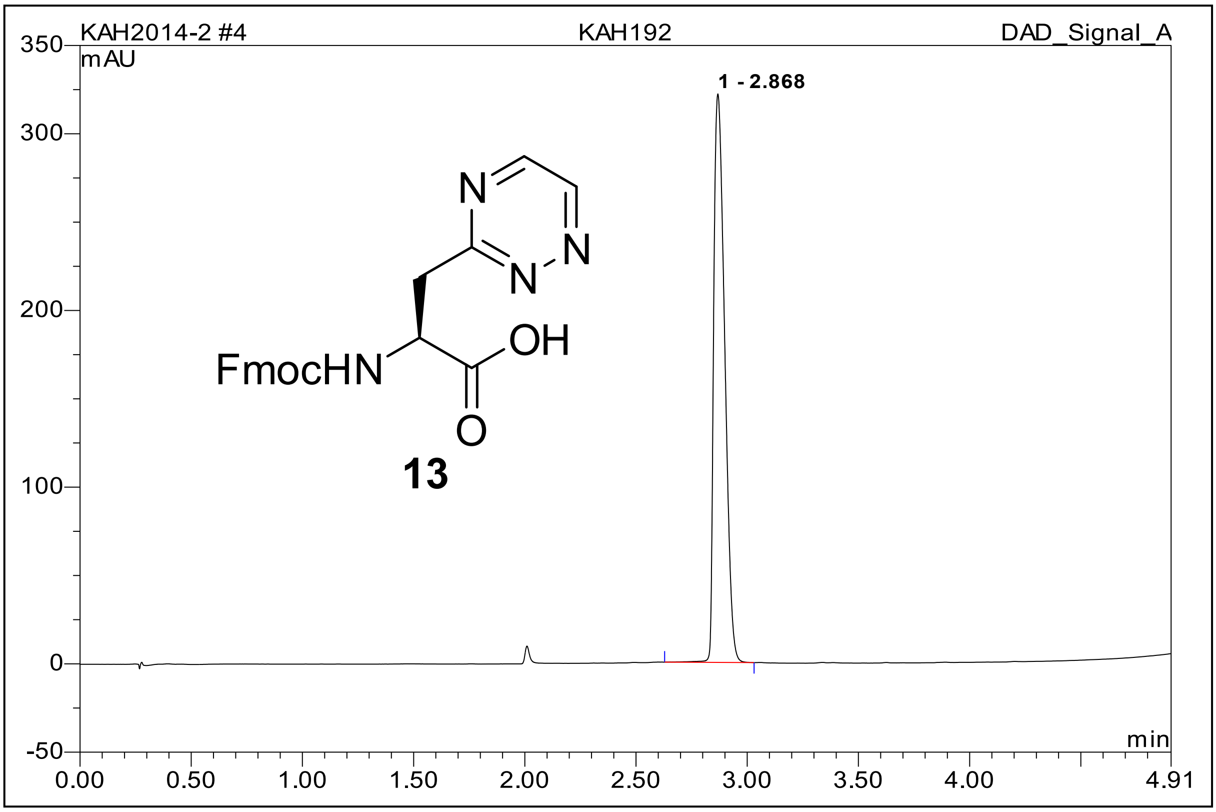

No.	Ret.Time min	Peak Name	Height mAU	Area mAU*min	Rel.Area %	Amount	Type
1	2.87	n.a.	321.804	18.444	100.00	n.a.	BMB
**Total:**			321.804	18.444	100.00	0.000	

School of Chemistry Mass Spectrometry Service

**SampleID**	KAH242	**Submitter**	Katherine Horner
**Sample Description**			
**Analysis Name**	KAH242_134994_BE2_01_12906.d	**Supervisor**	Stuart Warriner
**Method**	3c_AccMass_Loop_High_Pos.m	**Acquisition Date**	20/06/2015 11:26:23
**Instrument**	maXis impact **Source Type** ESI **Ion Polarity** Positive	**Scan Begin** 250 m/z	**Scan End** 3000 m/z

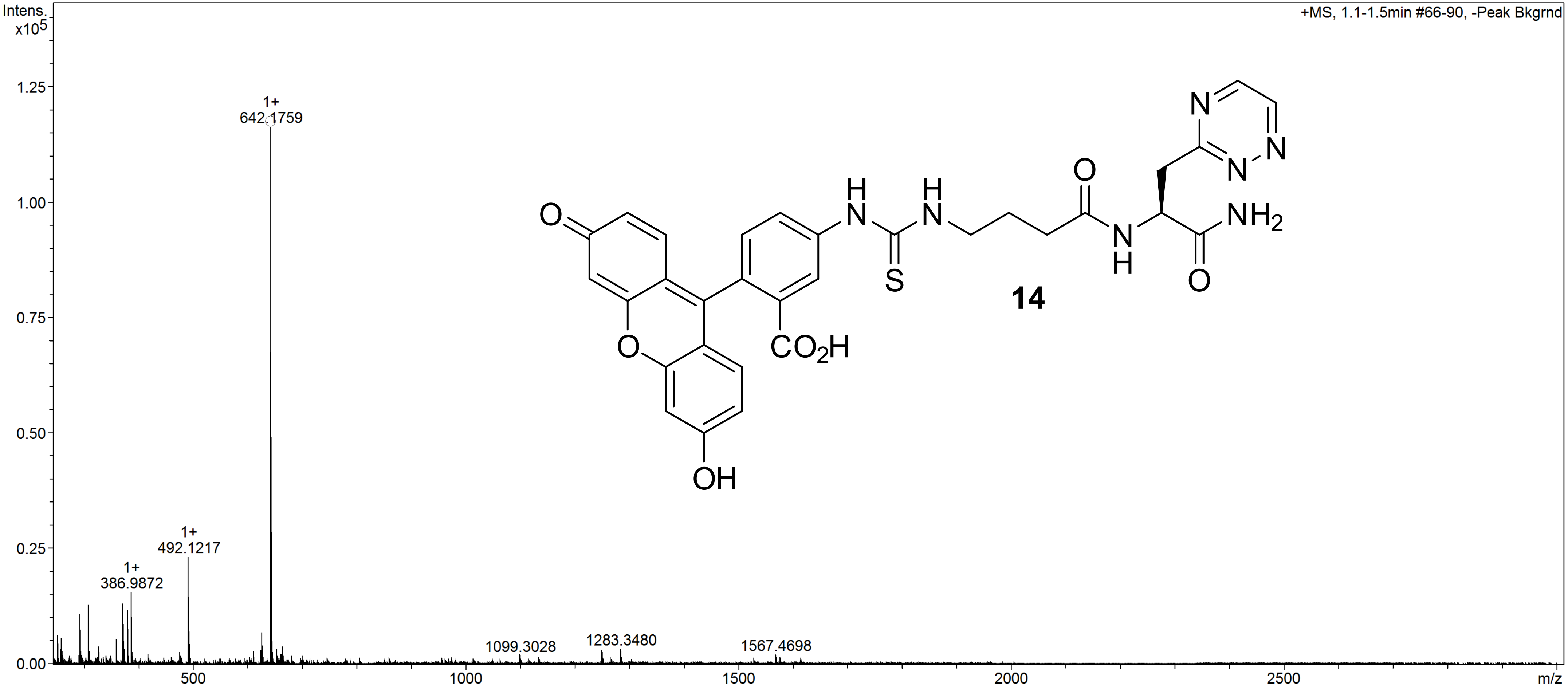

14

7 KAH242

Sample Name:	**KAH242**	*Injection Volume:*	**2.0**
Vial Number:	**P1:F6**	*Channel:*	**DAD_Signal**
Sample Type:	**unknown**	*Wavelength:*	**n.a.**
Control Program:	**Rapid5-95+TFA Ascentis Express C18**	*Bandwidth:*	**n.a.**
Quantif. Method:	**MartinH**	*Dilution Factor:*	**1.0000**
Recording Time:	**09/03/2015 12:29**	*Sample Weight:*	**1.0000**
Run Time (min):	**4.90**	*Sample Amount:*	**1.0000**

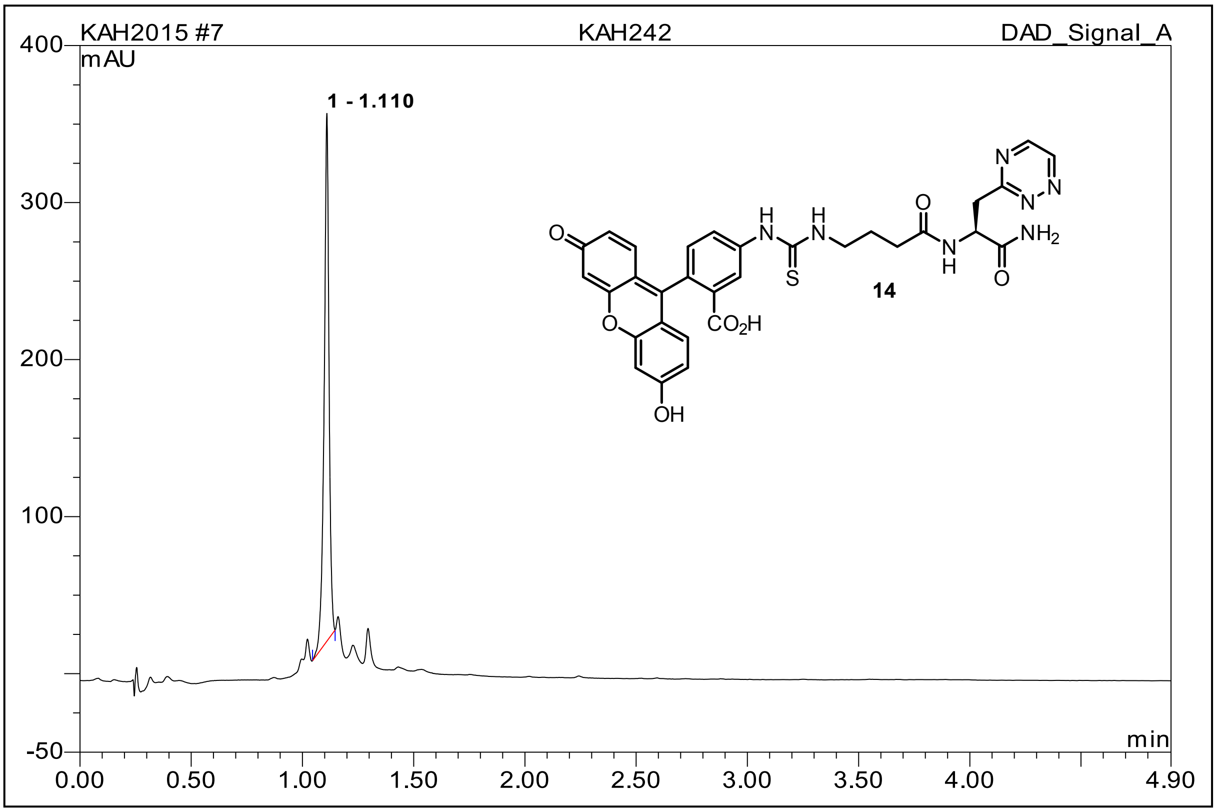

No.	Ret.Time min	Peak Name	Height mAU	Area mAU*min	Rel.Area %	Amount	Type
1	1.11	n.a.	336.214	8.533	100.00	n.a.	BMB
**Total:**			336.214	8.533	100.00	0.000	

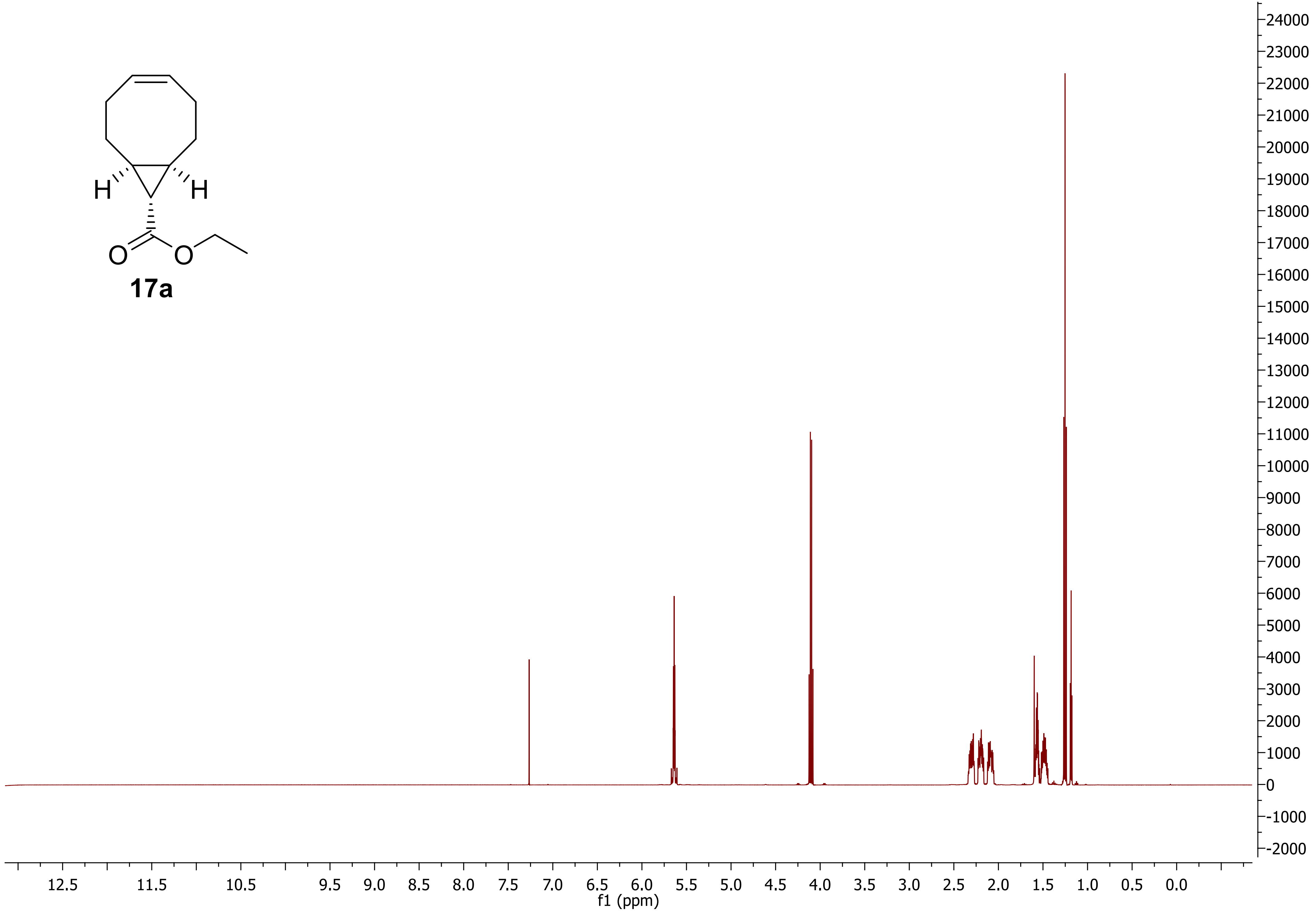
17a
f1 (ppm)
12.5
11.5
10.5
9.5
9.0
8.5
8.0
7.5
7.0
6.5
6.0
5.5
5.0
4.5
4.0
3.5
3.0
2.5
2.0
1.5
1.0
0.5
0.0
24000
23000
22000
21000
20000
19000
18000
17000
16000
15000
14000
13000
12000
11000
10000
9000
8000
7000
6000
5000
4000
3000
2000
1000
0
-1000
-2000

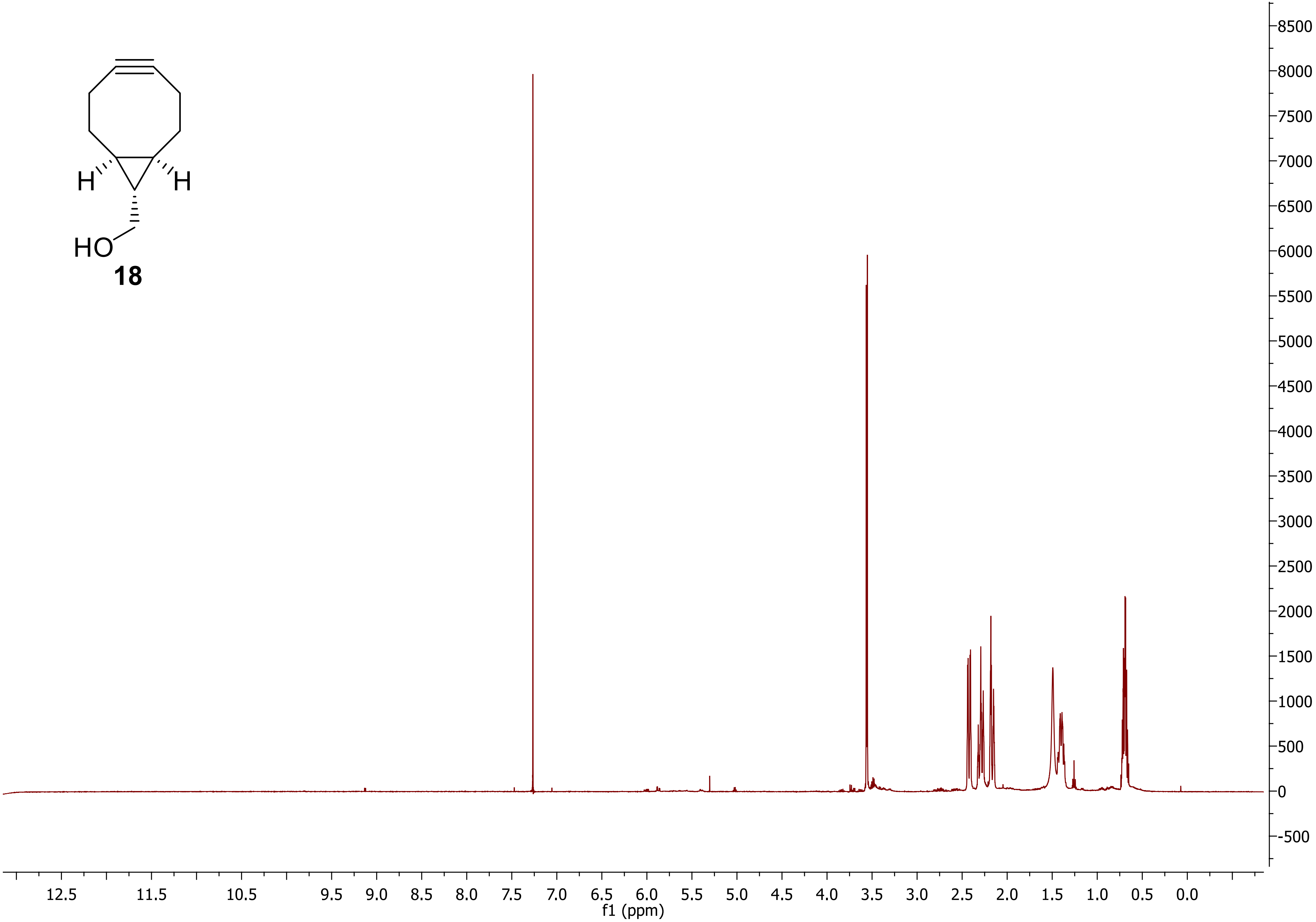
H
H
HO
18
8500
8000
7500
7000
6500
6000
5500
5000
4500
4000
3500
3000
2500
2000
1500
1000
500
0
-500
12.5
11.5
10.5
9.5
9.0
8.5
8.0
7.5
7.0
6.5
6.0
5.5
5.0
4.5
4.0
3.5
3.0
2.5
2.0
1.5
1.0
0.5
0.0
f1 (ppm)

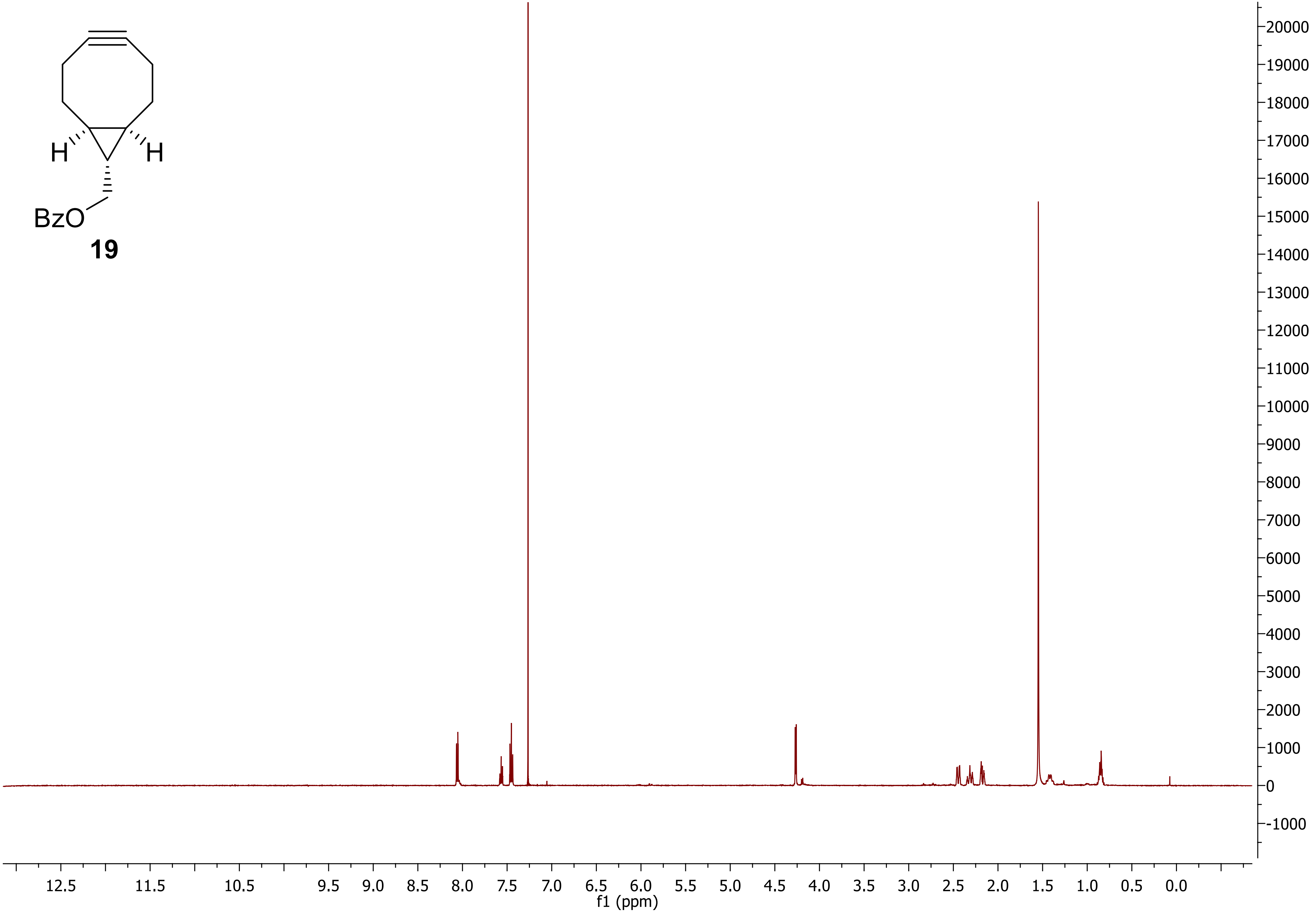
H
H
BzO
19
f1 (ppm)
12.5 11.5 10.5 9.5 9.0 8.5 8.0 7.5 7.0 6.5 6.0 5.5 5.0 4.5 4.0 3.5 3.0 2.5 2.0 1.5 1.0 0.5 0.0
20000 19000 18000 17000 16000 15000 14000 13000 12000 11000 10000 9000 8000 7000 6000 5000 4000 3000 2000 1000 0 -1000

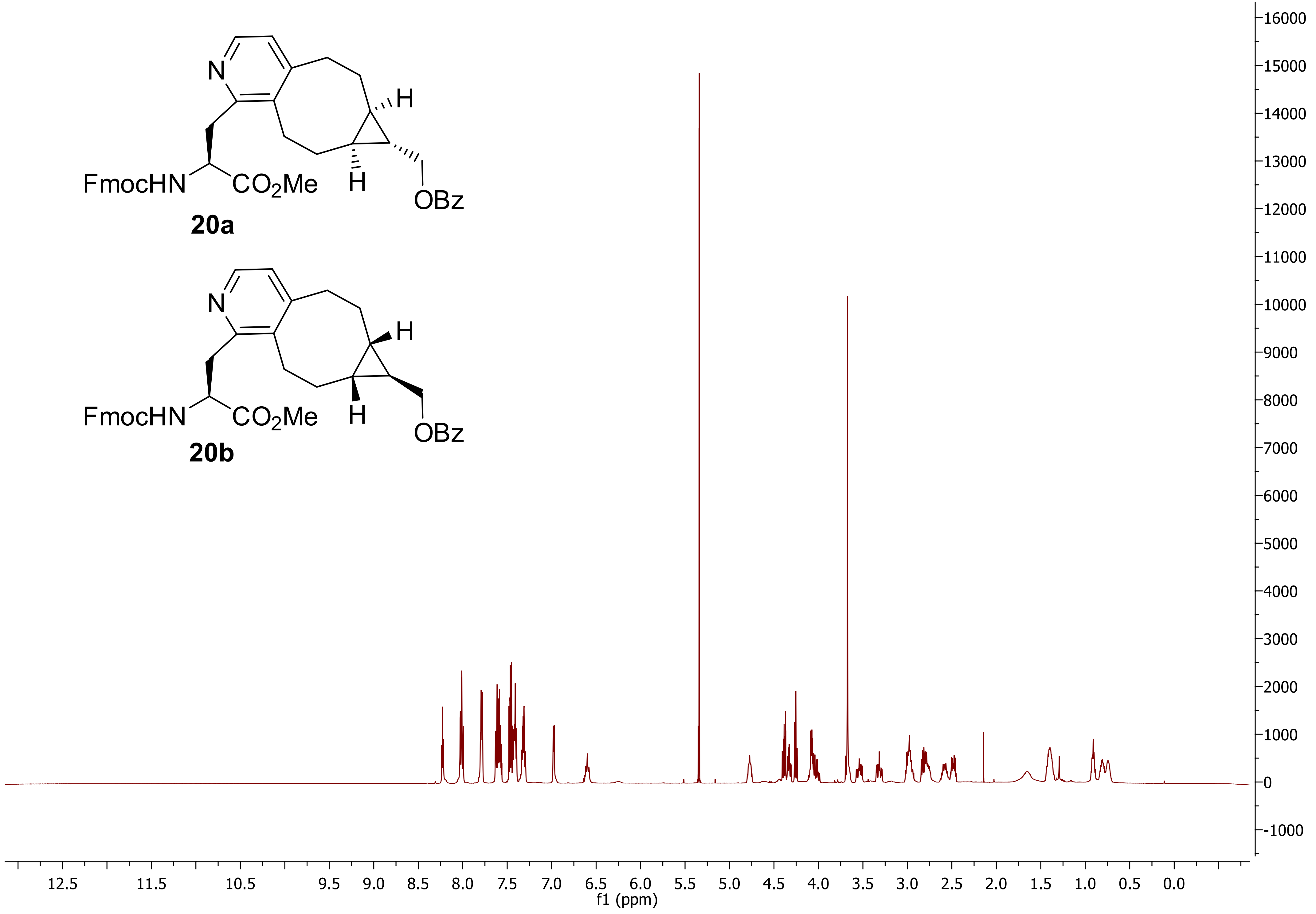

FmocHN
CO2Me
H
H
OBz
N
20a
FmocHN
CO2Me
H
H
OBz
N
20b
16000
15000
14000
13000
12000
11000
10000
9000
8000
7000
6000
5000
4000
3000
2000
1000
0
-1000
12.5
11.5
10.5
9.5
9.0
8.5
8.0
7.5
7.0
6.5
6.0
5.5
5.0
4.5
4.0
3.5
3.0
2.5
2.0
1.5
1.0
0.5
0.0
f1 (ppm)
